# Gene essentiality for tumour growth influences neoantigen‐directed immunoediting

**DOI:** 10.1002/ctm2.714

**Published:** 2022-01-21

**Authors:** Hyoeun Bang, Jae Soon Park, Jeong Yeon Kim, Changhwan Sung, Jinhyeon An, Dae‐Yeon Cho, Se‐Hoon Lee, Seok Bo Shim, Jung Kyoon Choi, Kwoneel Kim

**Affiliations:** ^1^ Department of Bio and Brain Engineering KAIST Daejeon Republic of Korea; ^2^ Penta Medix Co., Ltd. Seongnam‐si Gyeonggi‐do Republic of Korea; ^3^ Graduate School of Medical Science and Engineering KAIST Daejeon Republic of Korea; ^4^ Department of Health Sciences and Technology Samsung Advanced Institute of Health Science and Technology Sungkyunkwan University Seoul Republic of Korea; ^5^ Department of Biology Kyung Hee University Seoul Republic of Korea

Dear Editor,

The outgrowth of clones not expressing tumour antigens is a well‐known immune evasion mechanism of cancer.^1^ Here, we defined constitutive neoantigens according to the essentiality of their corresponding genes, which are essentially expressed for tumour growth even under immune selection. Hence, the essentiality of antigen‐producing genes may be an important determinant of neoantigen validity in cancer immunotherapy.

In our hypothesis, neoantigen‐producing genes that are initially expressed can be repressed in the process of cancer immunoediting when the corresponding neoantigens are targeted by the immune system (Figure S1).^2^, ^3^, ^4^ Neoantigens arising from essential genes, or constitutive neoantigens, will be less affected by this process and be presented consistently, resulting in clonal contraction.^5^, ^6^ Therefore, beneficial clinical responses are expected when many immunogenic neoantigens are derived from essential genes (Figure 1A). In contrast, dispensable genes that are not essential for the survival of the tumour generate facultative neoantigens that can be relevant to poor clinical responses (Figure 1B). Based on these hypotheses, we tested the influence of constitutive and facultative neoantigens using immune checkpoint inhibitor (ICI) cohort data and TCGA samples (Tables S1–S3) with leveraging data for the fitness effects of genes (Tables S4 and S5). Genes with high fitness effects in loss‐of‐function screens and with low heterogeneity in single‐cell transcriptome analyses were defined as essential (see Supporting Information).

**FIGURE 1:**
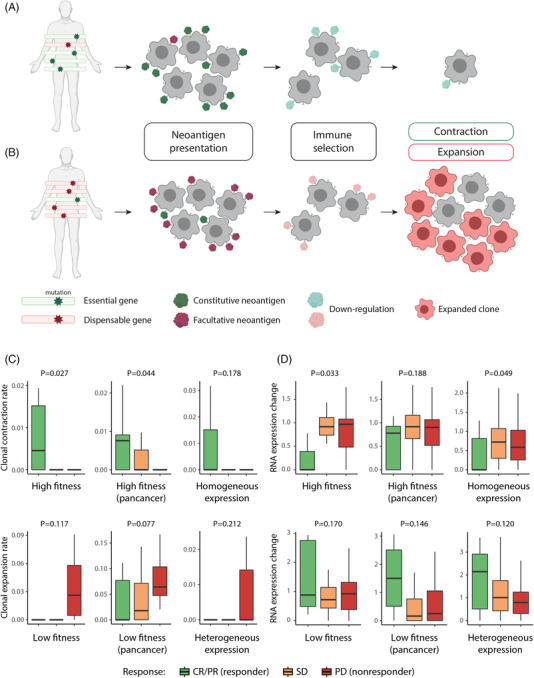
Association of cancer immunoediting with fitness effect of neoantigens. The constitutively presented neoantigens that are generated from essential cancer genes are likely to cause immune selection, resulting in tumour cell shrinkage (A). In contrast, facultatively presented neoantigens from dispensable cancer genes lead to tumour cell growth as a consequence of evading immune responses by repressing their expression (B). Comparison of clonal contraction rate among responses of patients in melanoma cell‐line‐specific high fitness (C, top left), pancancer high fitness (C, top middle), and homogeneously expressed (C, top right) gene groups. Comparison of clonal expansion rates in patients with different responses in melanoma cell‐line‐specific low fitness (C, bottom left), pancancer low fitness (C, bottom middle), and heterogeneously expressed (C, bottom right) gene groups. We used the top and bottom 500 genes with their fitness effect and heterogeneity ranks to define the gene groups of high/low fitness and homogeneous/heterogeneous expression. Comparison of RNA expression changes among patients with different responses in melanoma cell‐line‐specific high fitness (D, top left), pancancer high fitness (D, top middle), homogeneously expressed (D, top right), melanoma cell‐line‐specific low fitness (D, bottom left), pancancer low fitness (D, bottom middle), and heterogeneously expressed (D, bottom right) gene groups. All *p*‐values were computed by a Wilcoxon rank‐sum test between the CR/PR group and SD/PD group

We first examined whether clinical responses are associated with clonal and transcriptional changes in constitutive and facultative neoantigens because of immunoediting during ICI therapy. For this purpose, we employed data from a recent study on a melanoma cohort treated with anti‐PD‐1^7^ in both pre‐therapy and in‐therapy conditions. For responders, a larger proportion of immunogenic neoantigens may be derived from essential genes; thus, clonal reduction and transcriptional repression of constitutive neoantigens are expected. To test this, we measured clonal change by comparing pre‐therapy and in‐therapy mutation allele frequencies. We also normalized RNA expression levels by the number of DNA clones for each patient to compare expression changes (see Materials and Methods). Constitutive and facultative neoantigens were defined by the fitness effect and expression heterogeneity of the corresponding genes. As predicted by our hypothesis, the responders demonstrated significantly higher clonal contraction rate of constitutive neoantigens than non‐responders (Figure 1C, top and Figure S3, bottom) and consequently showed decreasing RNA expression of the constitutive neoantigens during treatment (Figure 1D, top), which could be interpreted as a result of immunoediting. In contrast, the non‐responders showed higher clonal expansion for mutations in genes with low fitness effects and heterogeneous expression than did the responders (Figure 1C, bottom and Figure S3, top). Clones with facultative neoantigens seemed to evade immune surveillance by immunoediting because their expression was downregulated during therapy in the non‐responders (Figure 1D, bottom).

Next, we employed a variety of patient cohorts treated with ICIs (Table S1). Overall, a high somatic tumour mutational burden (TMB) was associated with better overall survival (Figure 2A, left). Remarkably, the association between somatic TMB and overall survival was more significant when TMB was recalculated using essential genes (Figure 2A, middle). In contrast, the association between somatic TMB and overall survival was lower when the TMB of dispensable genes was used (Figure 2A, right). High neoantigen load supported better patient survival irrespective of the type of cancer and treatment, and this association was more significant when neoantigen load was measured from essential genes (Figure 2B,C and Figures S6, S7A, and S8A). Furthermore, neoantigen load estimated from essential genes showed better predictive power in classifying responders and non‐responders (Figure S9). These results imply that constitutive neoantigens arising from essential genes can be better predictors of clinical response to ICI than facultative neoantigens or all neoantigens.

**FIGURE 2:**
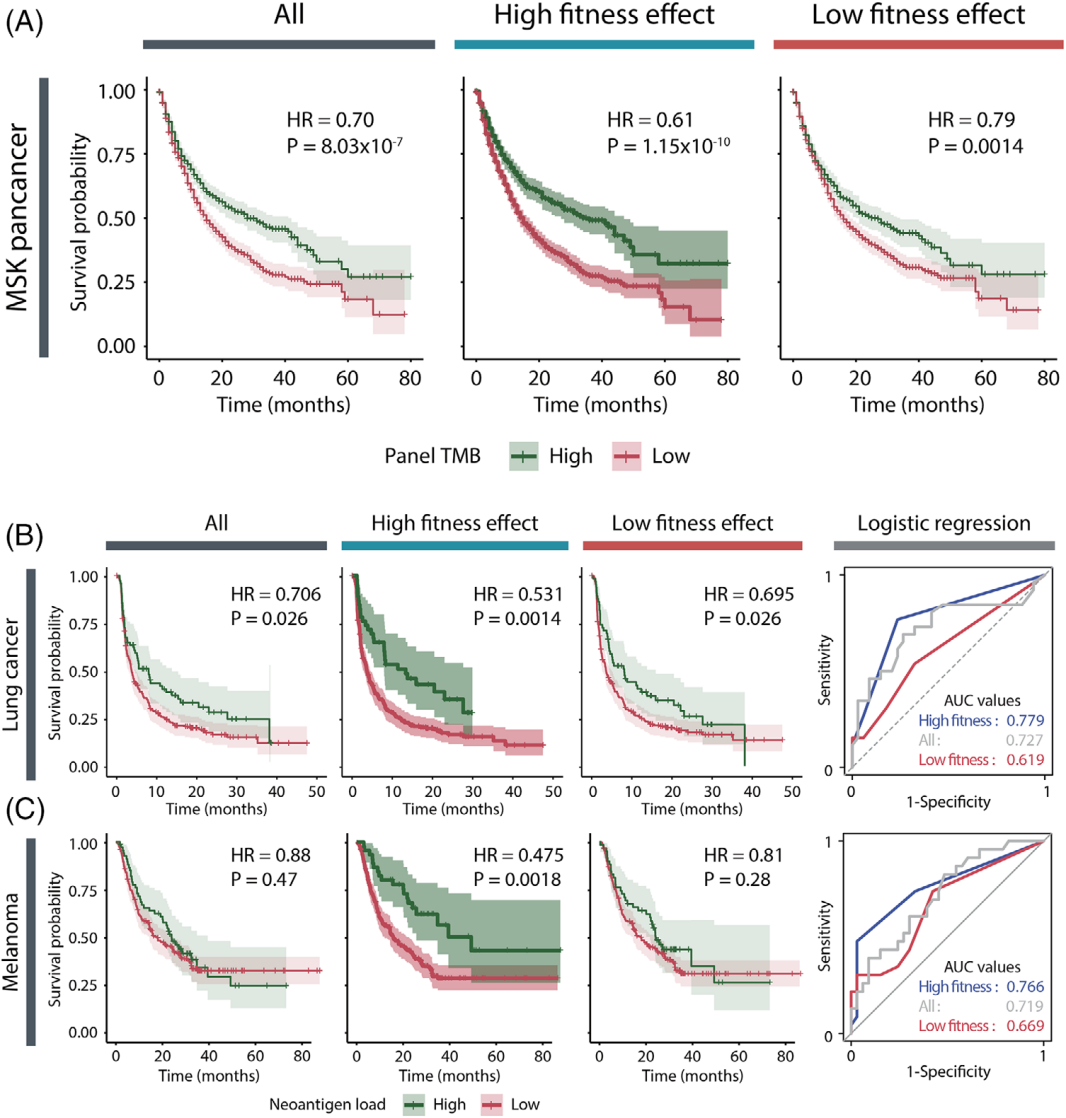
Survival analysis of immune checkpoint inhibitor (ICI) cohorts according to neoantigen load with fitness effects. Kaplan–Meier plot (KM plot) for overall survival (OS) of patients with high or low somatic tumour mutation burden of genes in MSK‐IMPACT panel (A, left), genes with a higher fitness effect than the median (A, middle), and genes with a lower fitness effect than the median (A, right). The KM plot for progression‐free survival (PFS) of all patients included multiple lung cancer ICI cohorts with high or low neoantigen load of all genes (B, left KM plot), the 500 genes with the highest ranked lung cancer‐specific fitness scores (B, middle KM plot) and the 500 genes with the lowest ranked scores (B, right KM plot). A logistic regression classifier was also used to predict ICI responses of lung cancer patients based on the three types of neoantigens (B, receiver operating characteristic curve; ROC curve). The odds ratios (OR) of the logistic regression for individual lung cancer cohorts are shown in Figure S9. The same analyses were repeated with OS (Figure 2C and Figure S9)

We further attempted to compare neoantigen load between responders and non‐responders while estimating neoantigen load from different numbers of genes with varying essentiality. In this analysis scheme, larger differences in neoantigen load imply greater power in segregating responders and non‐responders. Neoantigens were counted from 500–2000 genes with ranks of fitness effects, expression heterogeneity, and basal expression levels. Because different numbers of genes were used to estimate neoantigen load, we normalized the difference in the neoantigen count by the overall neoantigen count. As a result, larger normalized differences in neoantigen load were observed when genes with higher fitness effects or more homogeneous expression were considered (Figure 3A,B and Figures S7B, S8B, and S10). The trends were consistent regardless of the number of genes used for estimating neoantigens and the type of cancer. However, we observed no differences in differential neoantigen load based on basal expression levels (Figure 3C and Figure S11). Taken together, our results indicate that neoantigens assessed by fitness effects and expression heterogeneity can be a better determinants of cancer immunoediting than the basal level of neoantigen expression.

**FIGURE 3:**
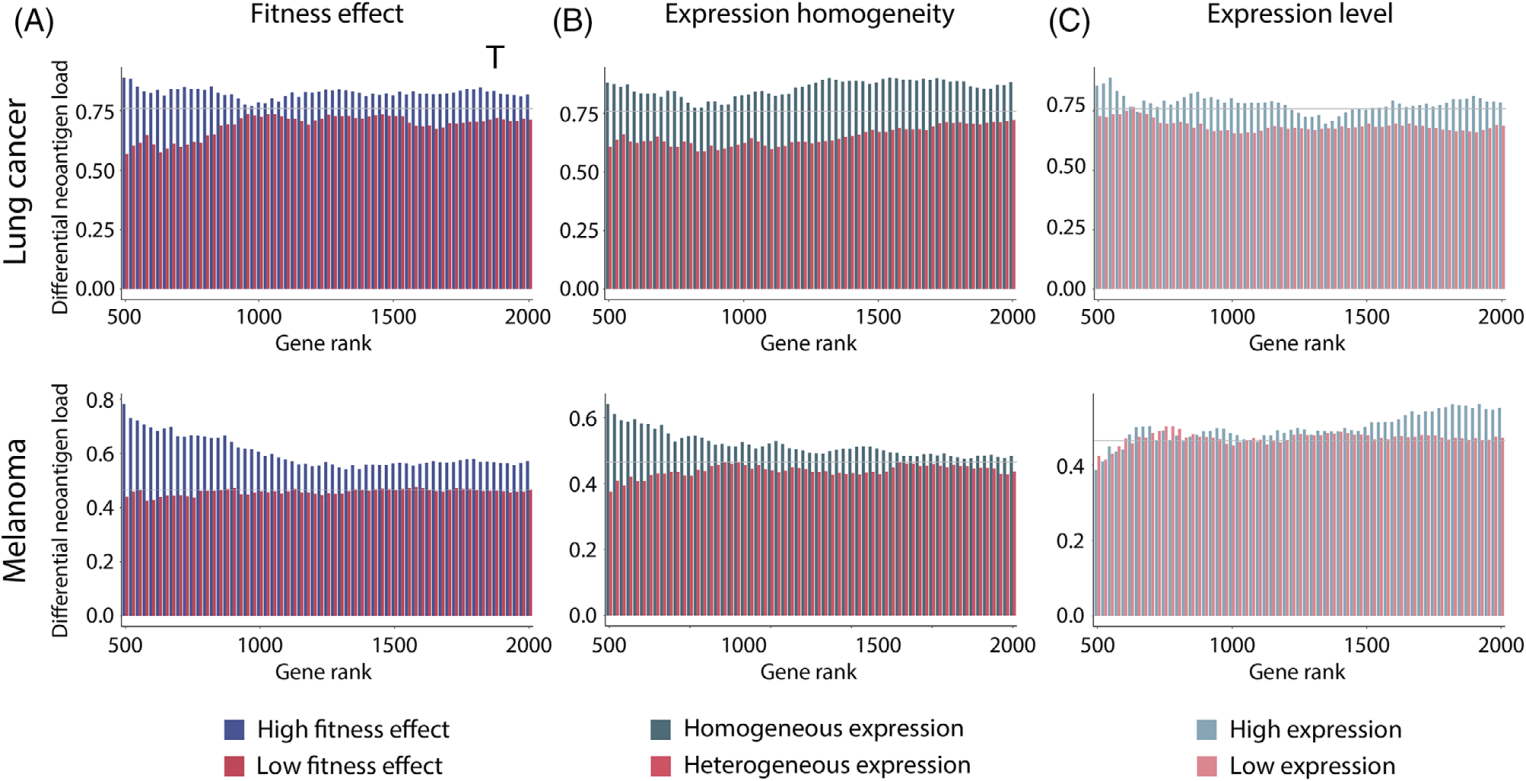
Comparison of differential neoantigen load with inclusion of increasing numbers of genes from fitness score, expression heterogeneity, and RNA expression value. Bar chart of differential neoantigen load in immune checkpoint inhibitor (ICI)‐treated lung cancer (top) and melanoma (bottom) cohorts. Comparison of differential neoantigen load in all genes (gray line in A), high fitness effect genes (blue bar in A), and low fitness effect genes (red bar in A). Associations between differential neoantigen load and expression heterogeneity (B) or RNA expression (C) are described by bar charts in the same manner as in (A). Gene rank indicates the ascending and descending ordered number of genes analyzed for fitness score, expression heterogeneity, and RNA expression

Finally, we validated the clinical implications of constitutive neoantigens using TCGA data. However, the association analysis between neoantigen load and therapeutic responses was not applicable because ICI therapy was not relevant in most TCGA samples. As a surrogate, we used the leukocyte fraction (LF)^8^ and immune cluster^9^ to simulate high immune selection because a dense immune cell population indicative of a high LF and hot‐tumor‐enriched (HTE) immune cluster is likely to trigger an antitumor immune response. However, if cancer cells can evade immune surveillance by immunoediting, the state of high LF and HTE would not lead to immune‐mediated patient survival. Under this rationale, we calculated the normalized differences in neoantigen load between long and short survivors with high LF and HTE. We focused on lung cancer and breast cancer because a deep neural network (DNN)‐based patient‐specific cancer fitness model^10^ was available for these cancer types. The constitutive and facultative neoantigens were defined by the predicted fitness effects (see Materials and Methods, Figure S12). Consistent with the ICI cohort results, larger differences in neoantigen load were observed when genes with high patient‐specific fitness effects were used for neoantigen counting (Figure 4A,B and Figures S13A, S13B, and S14). Remarkably, this pattern was observed in the high LF and HTE group but not in the low LF and coldtumour‐enriched group. This implies that patients with more constitutive neoantigens have a higher likelihood of long‐term survival due to immune‐mediated selection. This trend was more distinct when the DNN‐based patient‐specific model was used than when cell line screening data were used (Figure 4C,D and Figure S13C,D). The DNN model predicted the fitness effect of genes for each patient using the corresponding RNA‐seq profile. Therefore, patient‐specific constitutive neoantigens seem to explain survival better than non‐specific constitutive neoantigens derived from cell line screening.

**FIGURE 4:**
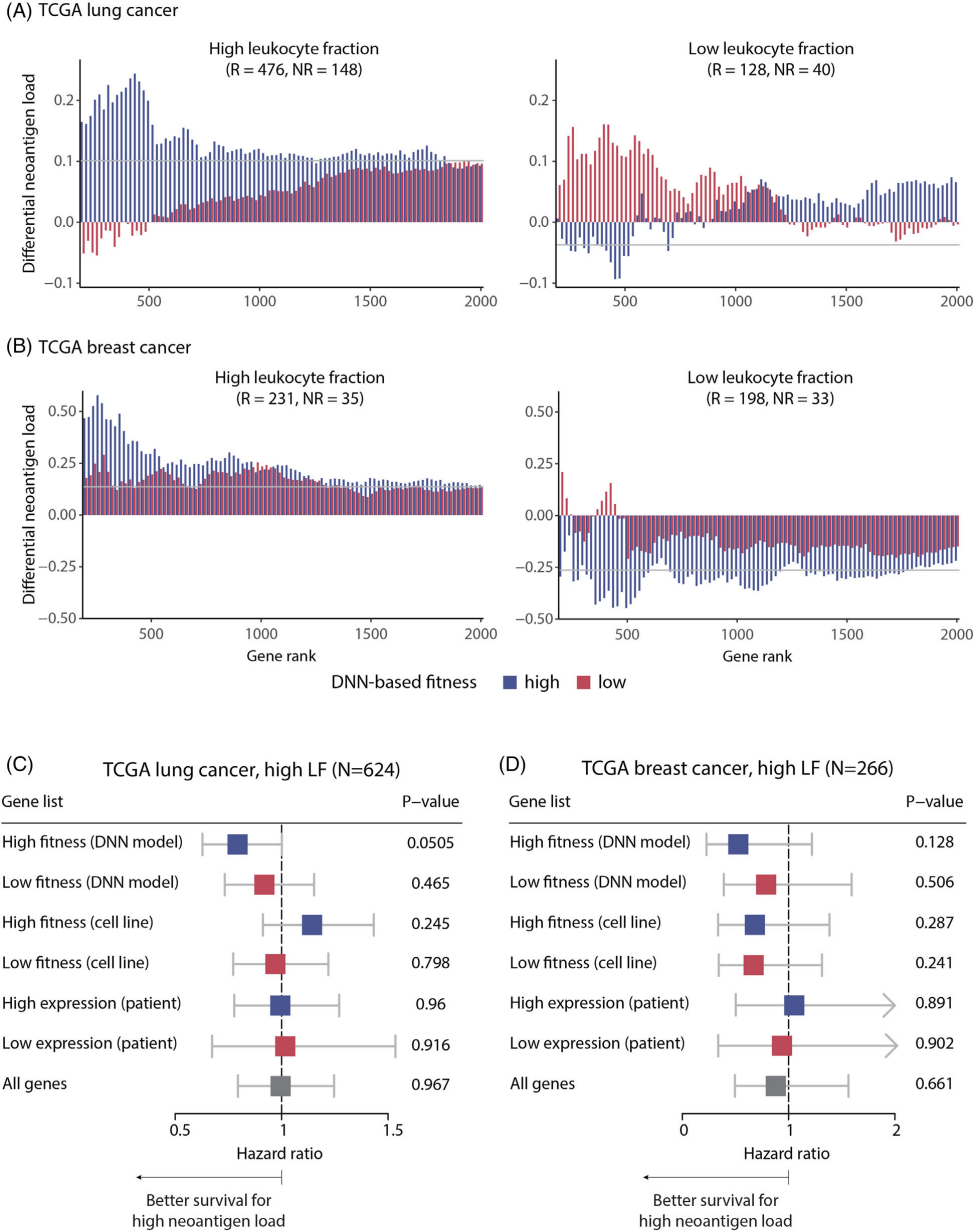
Survival analysis of TCGA patients based on deep neural network (DNN)‐estimated patient‐specific fitness effects. Bar chart of differential neoantigen load between long‐term and short‐term survivors of TCGA lung cancer and breast cancer patients with high leukocyte fraction (LF) (A and B, left) and low LF (A and B, right). The fitness effect of neoantigens was defined based on the score predicted by the DNN model. The 200–2000 genes with the highest and lowest DNN‐estimated vulnerability of each patient were used to calculate the differential neoantigen load. Forest plot demonstrating hazard ratios for overall survival according to 500 genes with highest and lowest fitness scores from DNN model prediction, cell‐line‐based screening, and patient‐specific basal expression in TCGA lung cancer (C) and breast cancer patients (D)

In this work, we discovered novel determinants of neoantigen‐directed immunoediting that can be used to evaluate the clinical value of neoantigens, particularly in the setting of ICI treatment. Our results explicitly suggest that neoantigens derived from essential genes have the potential to minimize immune evasion and contribute to favourable responses to ICI therapy. Further investigation will be needed to provide underlying mechanisms of constitutive neoantigen‐directed immunoediting.

## CONFLICT OF INTEREST

The authors declare no conflict of interest.

## Supporting information

Supporting Information

Supplementary Table 6. Values of expression heterogeneity in lung cancer

Supporting Information
