# Supplementary material for: Gene essentiality for tumour growth influences neoantigen‐directed immunoediting
Source: Clin Transl Med. 2022 Jan 21;12(1):e714. doi: 10.1002/ctm2.714 (PMC8778643; doi:10.1002/ctm2.714)
Supplement: Supplementary file 1 — Supporting Information [file CTM2-12-e714-s002.docx]

**Gene essentiality for tumor growth influences neoantigen-directed immunoediting**

**Materials and methods**

**Cohort information**

We employed a variety of patient cohorts treated with ICIs (Table S1). The first cohort included 1,662 advanced cancer samples that were sequenced by MSK-IMPACT panel (pancancer cohort). The same analyses for the pancancer cohort were performed by combining three lung cancer ICI cohorts and four melanoma ICI cohorts that included whole-exome sequences. Most samplings for ICI cohorts were performed at pre-treatment baseline except the Rizvi cohort in which both pre- and on-therapy data were available.

| Cohort | Tumor type | Target checkpoint | Cohort size | Biopsy time | Histology (lung) /  Primary (melanoma) |
| --- | --- | --- | --- | --- | --- |
| MSKCC [1] | Pancancer | PD-1, CTLA-4 | 1662 | N/A | N/A |
| Shim [2] | Lung cancer | PD-1/PD-L1 | 197 | pre (197) | LUAD (128), LUSC (58), others (11) |
| Hellmann [3] |  | PD-1, CTLA-4 | 75 | pre (74), on (1) | LUAD (59), LUSC (16) |
| Rizvi [4] |  | PD-1 | 34 | pre (33), on (1) | LUAD (29), LUSC (4), NSCLC NOS (1) |
| Van Allen [5] | Melanoma | CTLA-4 | 110 | pre (110) | Cutaneous (92), occult (14), mucosal (4) |
| Riaz [6] |  | PD-1 | 68 | pre (68) | Cutaneous (46), acral (2), mucosal (7), ocular/uveal (5), others (13) |
| Snyder [7] |  | CTLA-4 | 64 | pre (34), post (30) | Cutaneous (26), acral (2), uveal (1), occult (3),  not available (7) |
| Hugo [8] |  | PD-1 | 38 | pre (34), on (4) | N/A |
| Mariathasan [9] | Bladder cancer | PD-L1 | 216 | pre (216) | N/A |
| McDermott [10] | Renal cell carcinoma | PD-L1 | 50 | pre (50) | N/A |
| TCGA | Lung cancer | N/A | 519/497 | N/A | LUAD (519), LUSC (497) |
|  | BRCA | N/A | 1097 | N/A | N/A |

Table S1. Summary of ICI and TCGA cohorts

**Clinical benefit of cohorts**

We defined the clinical benefit using RECIST criteria and progression-free survival (PFS) or overall survival (OS). In lung cancer cohorts, a “responder” was defined as a patient with complete response (CR), partial response (PR) or stable disease (SD) with PFS greater than 6 months, whereas a “nonresponder” was defined as a patient with progressive disease (PD) or SD with PFS less than 6 months. In melanoma cohorts, a “responder” was defined by CR, PR or SD with OS greater than 1 year, and a “nonresponder” was defined by PD or SD with OS less than 1 year.

| **Cohort** | **Tumor type** | **Number of patients** | | | | | |
| --- | --- | --- | --- | --- | --- | --- | --- |
|  |  | **Total** | **RECIST** | | | **Clinical benefit** | |
|  |  |  | **CR/PR** | **SD** | **PD** | **Responder** | **Non**  **responder** |
| **Shim** | Lung cancer | 197 | 61 | 52 | 84 | 74 | 121 |
| **Hellmann** |  | 75 | 24 | 27 | 17 | 37 | 33 |
| **Rizvi** |  | 34 | 12 | 10 | 12 | 19 | 14 |
| **VanAllen** | Melanoma | 110 | 17 | 12 | 76 | 27 | 83 |
| **Riaz** |  | 68 | 15 | 23 | 27 | 31 | 35 |
| **Snyder** |  | 64 | 19 | 16 | 29 | 33 | 31 |
| **Hugo** |  | 38 | 21 | 0 | 17 | 21 | 17 |
| **Mariathasan** | Bladder cancer | 216 | 53 | 44 | 119 | 53 | 163 |
| **McDermott** | Renal cell carcinoma | 50 | 11 | 24 | 15 | 17 | 23 |

Table S2. Clinical information of ICI cohorts

**Exome alignment and somatic variant calling**

We collected the raw exome sequencing data of Shim, Hugo, Mariathasan, and McDermott cohorts. The sequencing reads from the tumor and matched normal samples were aligned to the hg19 human genome build using Burrows-Wheeler aligner (BWA; version 0.7.17-r1188) [11], which was followed by standard preprocessing steps including the marking of duplicate reads by Picard software (version 2.25.0; http://broadinstitute.github.io/picard/) and the recalibration of base quality by GATK-4.1.6.0 [12]. Somatic variants were detected with GATK4 Mutect2 [13] from the matched bam files based on the panel of normal created by the matched normal samples.

**Neoantigen prediction**

Four-digit class I HLA types were first determined using POLYSOLVER [14]. We computed predictive neoantigens by NetMHCpan-4.0 [15] from somatic variants and HLA types, which can be publicly accessed in the Shim, Hellmann, Hugo, Mariathasan, and McDermott cohorts. In the Rizvi, Van Allen, Riaz, and Snyder cohorts, we used the list of neoantigens from publications. We used only 9-mer neoepitopes derived from missense mutations, excluding deleterious mutations identified with the SIFT 4G annotator [16] (GRCh37.74).

| Cohort | Tumor type | HLA typing | Neoantigen prediction |
| --- | --- | --- | --- |
| Shim | Lung cancer | POLYSOLVER | NetMHCpan-4.0 |
| Hellmann |  | OptiType | NetMHCpan-4.0 |
| Rizvi |  | HLA-SBT (Invitrogen)  ATHLATES | NetMHC v3.4 |
| Van Allen | Melanoma | POLYSOLVER | NetMHCpan v2.4 |
| Riaz |  | SOAP-HLA | NetMHC-4.0 |
| Snyder |  | HLA-SBT (Invitrogen)  ATHLATES | NetMHC v3.4 |
| Hugo |  | ATHLATES | NetMHCpan-4.0 |
| Mariathasan | Bladder cancer | POLYSOLVER | NetMHCpan-4.0 |
| McDermott | Renal cell carcinoma | POLYSOLVER | NetMHCpan-4.0 |
| TCGA | LUAD/LUSC | OptiType | NetMHCpan v3.0 |
|  | BRCA |  |  |

Table S3. Method of HLA typing and neoantigen prediction for each cohort

**Fitness effect estimation**

We obtained dependency results from CRISPR-Cas9 knockout and RNAi screens in a range of cancer cell lines. We used the largest CRISPR-Cas9 screening resources [17], spanning 786 different cell lines of 42 cancer types, which combine two independent datasets generated at the Broad [18, 19] and Sanger institutes [20]. The data were preprocessed using the CERES scoring approach, batch-corrected, and adjusted to fold-change depletion values. In addition, we obtained RNAi dependency metrics covering 712 cancer cell lines combined from 3 large datasets – Project Achilles [21] (Broad Institute), Project DRIVE [22] (Novartis), and Marcotte *et al*. [23] – which were preprocessed using the DEMETER2 model [24].

Since both dependency metrics – CERES-FC from CRISPR and DEMETER2 from RNAi – appear to be correlated with each other (Figure S2A, S2B, and S2C), we integrated both metrics into a fitness score for each tumor type, including lung cancer, melanoma, and breast cancer. Both metrics were cellwise averaged, sign-reversed, min-max scaled separately for positive and negative values and averaged into the fitness score; a score of 1 indicates a high fitness effect, and a score of -1 indicates a low fitness effect. We used only protein-coding genes and excluded the genes that were present in the RNAi dataset only (Figure S2D). We further checked the consistencies between the CRISPR and RNAi screens (Figure S3).

We also investigated the ADaM core essential genes [20] (n=553), which have high fitness effects across all tumor types, and gold-standard nonessential genes [25] (n=927), which have low fitness effects across different cancer types. Additionally, we employed core fitness effect genes identified across different tumor types [26] and the curated gold-standard nonessential genes [27].

| Knockout screens | | CRISPR-Cas9 | RNAi |
| --- | --- | --- | --- |
| Algorithm | | **Unified CERES-FC** [17] | **DEMETER2** [24] **combined (v6)** |
| Combined datasets | | Project Achilles (Broad)  Project SCORE (Sanger) | Project Achilles (Broad)  Project DRIVE (Novartis)  Marcotte *et al*. |
| Cancer type | Lung cancer | 107 | 127 |
|  | Melanoma | 45 | 46 |
|  | Bladder cancer | 27 | 12 |
|  | Renal cell carcinoma | 24 | 27 |
|  | Breast cancer | 39 | 79 |
|  | Pancancer | 786 | 712 |

Table S4. Number of cancer cell lines in the CRISPR and RNAi knockout screening datasets used for analysis

| Knockout screens | | CRISPR-Cas9 | | RNAi | | |
| --- | --- | --- | --- | --- | --- | --- |
| Algorithm | | **Unified CERES-FC** | | **DEMETER2_v6 combined** | | |
| Dataset | | Achilles | SCORE | Achilles | DRIVE | Marcotte *et al*. |
| Cancer type | Lung cancer | 78 | 36 | 115 | 63 | 0 |
|  | Melanoma | 42 | 4 | 37 | 36 |  |
|  | Bladder cancer | 27 | 0 | 3 | 12 |  |
|  | Renal cell carcinoma | 22 | 3 | 24 | 16 |  |
|  | Breast cancer | 29 | 26 | 34 | 25 | 76 |
|  | Pancancer | 625 | 319 | 501 | 397 | 76 |

Table S5. Number of cancer cell lines in individual datasets used to produce aggregate CERES-FC and DEMETER2 scores.

**Expression heterogeneity**

At the organismal level, it has been suggested that expression variation, rather than expression level, is an indicator of the essentiality and evolutionary rate of genes [28]. This concept can also be applied to a population of cells. To perform core functions in the cell, essential proteins may be consistently required among individual cells with low cell-to-cell variation. In fact, it has been shown that proteins with high fitness effects exhibit minimal levels of cell-to-cell expression noise [29, 30]. We adapted this concept to cancer cell populations and used the intratumor heterogeneity of gene expression as estimated by single-cell transcriptomes [31–34] to identify constitutive and facultative neoantigens.

Expression heterogeneity was calculated based on the single-cell RNA expression profiles of 17,061 lung cancer epithelial cells [35] and 7,186 cells derived from 33 melanoma tumor tissues [36]. We used genes with nonzero UMIs greater than 95% cell coverage in lung cancer and protein-coding genes [37] with an average TPM>=0.1 in melanoma. We calculated the coefficient of variation (CV) for each gene as shown in Equation 1:

$$Coefficient of Variation \left( CV \right)= \frac{Standard Deviation (\sigma)}{Average (\mu)}$$

(Equation 1)

To obtain cell-to-cell gene expression divergence, independent of expression level, the CV of each gene was compared with the CV of control genes: 1,000 genes randomly selected among the genes that have a mean TPM within twofold of the expression levels of the gene of interest [38]. The expression heterogeneity was determined from Equation 2:

$$Expression Heterogeneity= \frac{CV of the gene of interest}{average CV of control genes}$$

(Equation 2)

Expression heterogeneity in the lung cancer and melanoma sampleswas then compared to fitness effects in the matched tumor type. Although the correlation was marginal, lower intratumoral expression heterogeneity tended to show higher fitness effects (Figure S4). We then tested the association between neoantigen load and patient survival in the context of expression heterogeneity. Consistent with the results based on fitness effects, greater power in predicting survival benefit by neoantigen load was observed when neoantigens derived from homogenously expressed genes were counted in contrast to when neoantigens from heterogeneously expressed genes were counted (Figure S5). We summarized the fitness effect and expression heterogeneity of each gene for each cancer type in Table S6. The high scoring genes were mostly involved in housekeeping function such as translation, nuclear transport, protein regulation, and splicing regulation, distinguishing themselves from oncogenes or tumor suppressors that promote uncontrolled cell proliferation in association with driver mutations.

**Clonal expansion and contraction**

We examined the association of mutational expansion or contraction rate with gene essentiality and intratumor expression heterogeneity using the previously estimated cancer cell fraction (CCF) [6, 39] – the fraction of tumor cells carrying a mutation. Mutations whose CCF increased or decreased by more than 40% from pre- to in-therapy samples were considered expanded or contracted clones. The proportions of mutations in the gene sets within expanded or contracted clones were analyzed according to RECIST criteria.

**RNA expression change**

$$RNA expression change= \frac{(\frac{FPKM of mutated gene in-therapy}{FPKM of mutated gene pre-therapy})}{(\frac{CCF of mutation in-therapy}{CCF of mutation pre-therapy})}$$

(Equation 3)

For each gene with missense mutations, RNA expression changes from pre-therapy to in-therapy, normalized by CCF, were calculated as shown in Equation 3. The formula calculates the normalized change in the expression level of genes harboring mutations during immunotherapy.

**Survival analysis of the MSK-IMPACT cohort and ICI cohorts**

Since a total of 412 genes had fitness scores in 468 MSK-IMPACT panels, 206 genes with fitness scores greater than the median were selected as having higher fitness, and another 206 genes were selected as having lower fitness. The patients were classified by the median value of each counted TMB. Progress-free survival was analyzed in lung cancer cohorts, and overall survival was analyzed in melanoma cohorts. The patients were classified by the 3^rd^ quantile value of each counted neoantigen load in each cohort.

**The calculation of differential neoantigen load**

$$Differential Neoantigen Load=\frac{Mean \left( NeoAg_{Responder} \right)-Mean\left( NeoAg_{Nonresponder} \right)}{Mean(NeoAg_{All})}$$

(Equation 4)

where:
NeoAg_Responder_ = Neoantigen load of the responders
NeoAg_Nonresponder_ = Neoantigen load of the nonresponders
NeoAg_All_ = Neoantigen load of all patients.

The differential neoantigen load for the gene sets of high or low fitness effect genes and all genes was calculated as shown in Equation 4. For high fitness effect genes (blue bars in Figure 3), we counted the neoantigens from the top *n* high fitness effect genes in the responder and nonresponder groups. Subsequently, we divided the difference in neoantigen counts between the two groups by the average counts of the top *n* high fitness effect genes in all patients. The normalization by dividing with the neoantigen load of all patients was needed to compare with other differential neoantigen loads that were calculated using different *n* genes. We then repeated the calculations with a larger number of high or low fitness effect genes and overall genes.

**TCGA cohort RNA expression**

All genes were ordered according to the sample-wise mean of RNA-seq FPKM in the pancancer, LUAD, LUSC, and SKCM cohorts to test the association between ICI benefits and basal expression levels of neoantigens (Figure 3C and S9). The lung cancer cohorts were analyzed by samplewise mean in the combined cohorts of both LUAD and LUSC. All RNA-seq TCGA datasets are publicly available on the UCSC Xena platform[40]. We speculated that the basal expression level of a gene should not be considered to be crucial in ascertaining gene essentiality because essential proteins would be expressed with spatiotemporal consistency rather than be expressed at high concentrations. To corroborate this idea, we first compared the survival of samples with high neoantigen load versus that of samples with low neoantigen load while estimating neoantigen load from genes with high or low expression levels. We selected 500 genes with the highest or lowest expression levels in the matched cancer types. As a result, we observed no differences in the degree of the segregation of survival between genes with high versus low expression levels consistently across different cancer types (Figure S9).

**Estimation of patient-specific fitness effects**

We employed the deep neural network (DNN)-based patient-specific fitness prediction model for breast cancer from our previous works [41, 42]. In addition, we constructed a predictive model for lung cancer using the same algorithm with CRISPR-Cas9 dropout screens of lung cancer cell lines. The test AUC of the DNN model was 86.2 (Figure S10), and the detailed process is described below. We downloaded CERES score-based CRISPR-Cas9 dropout screening results for 70 lung cancer cell lines [18] and their gene expression profiles from https://depmap.org/portal/download. Genes with CERES scores less than -1.5 were selected as genes with a high fitness effect, and a similar number of genes with CERES scores close to 0 were selected as neutral genes. We applied ARACNe-AP (Algorithm for the Reconstruction of Accurate Cellular Networks with Adaptive Partitioning) software [43] to construct a lung cancer regulatory network. The software is available at https://github.com/califano-lab/ARACNe-AP. We downloaded 1,129 lung cancer (576 LUAD and 553 LUSC) TCGA RNA-seq datasets from UCSC Xena[40] to infer direct transcriptional interactions based on mutual information (MI). We selected MI with P < 0.05. To determine the direction and regulation type of the interactions derived from ARACNe, a conditional probability-based method from our previous work [42] was used. Using the constructed regulatory network, the effects of CRISPR perturbation on RNA expression in lung cancer cell lines were simulated. The expression data simulating perturbations of high fitness effect genes were set as true cases, and neutral gene perturbations were set as false cases for deep neural network modeling. We constructed 1,000 models with random parameter combinations and then selected the deep neural network (DNN) model with the highest AUC in the validation dataset. The detailed method and explanations are provided in our previous work [42].

**Immune clustering**

We inferred the abundance of 64 cell types for TCGA lung cancer and breast cancer patients by transcriptomic signatures using XCell [44]. The transcriptomic signatures were then normalized by z-scores, and consensus clustering was performed to identify patients’ immune clusters that were hot tumor enriched and cold tumor enriched. Consensus clustering was implemented using the R package ConsensusClusterPlus [45].

**Supplementary figure legends**

**Figure S1.** Cancer cells with somatic mutations present neoantigens and are targeted by immune cells so that they regress. As a result of this cancer immunoediting, only the cells that repress the neoantigen-producing genes survive and consequently expand.

**Figure S2.** CRISPR and RNAi metrics are mutually significantly correlated and appropriate to integrate. CERES-based CRISPR-Cas9 depletion scores and D2-estimated RNAi dependencies were correlated with each other in lung cancer cell lines (A), melanoma cell lines (B), and pancancer cell lines (C). Venn diagram of the counts of protein-coding genes based on the CERES scores of CRISPR-cas9 knockout screens and DEMETER2 scores of RNAi screens (D).

**Figure S3.** Comparison of clonal expansion rate among patients with different responses in the melanoma cell-line-specific high fitness effect (top left), pancancer high fitness effect (top middle) and homogeneously expressed (top right) gene groups. Comparison of clonal contraction rate among patients with different responses in the melanoma cell-line-specific low fitness effect (bottom left), pancancer low fitness effect (bottom middle) and heterogeneously expressed (bottom right) gene groups.

No differences were expected between responders and nonresponders in clonal expansion of constitutive neoantigens and clonal contraction of facultative neoantigens. When assessed in relation to clinical responses, clones bearing constitutive and facultative neoantigens should show differences only in clonal contraction and expansion, respectively. As expected, constitutive neoantigens did not show a significant difference in clonal expansion between the responders and nonresponders (upper). Likewise, we observed no significant difference in clonal contraction of facultative neoantigens between the responders and nonresponders (lower). All p-values were computed by Wilcoxon rank-sum test between CR/PR group and SD/PD group.

**Figure S4.** Correlation between fitness effect and expression heterogeneity.

The expression heterogeneity (from Equations 1 and 2) and fitness effect are correlated according to the Spearman correlation test: Cor = -0.087, P-value = 5.651e-18 in lung cancer (A); Cor = -0.173, P-value = 8.335e-71 in melanoma (C). Box plots of expression heterogeneity according to the top 200, 500, 1000, and 2000 ranked high fitness effect and low fitness effect genes in lung cancer (B) and melanoma (D) are shown. *** indicates p < 1.00e-3 (Wilcoxon rank-sum test).

**Figure S5.** Survival analysis of ICI cohorts according to expression heterogeneity.

The KM plot for progression-free survival (PFS) of all patients in multiple lung cancer ICI cohorts according to high or low neoantigen load of the top 500 genes ranked according to homogenous expression (top left) and heterogeneous expression (top right) in lung cancer. The KM plot for overall survival (OS) of melanoma ICI patients according to homogenously expressed (top left) and heterogeneously expressed genes (top right).

**Figure S6.** Forest plot showing hazard ratios for progression-free survival (PFS) or overall survival (OS) corresponding to the 500 genes with the highest and lowest fitness effect scores in individual ICI cohorts of lung cancer (A) and melanoma (B). They were also analyzed divided by the treatment statement in lung cancer (C) and melanoma (D),

**Figure S7.** Comparison of differential neoantigen load and patient survival based on fitness scores of genes in the ICI-treated bladder cancer cohort.

The KM plot for overall survival (OS) of bladder cancer ICI patients [9] according to high fitness (A, middle), low fitness (A, right), and all genes (A, left). The fitness genes were obtained based on the top 1000 or bottom 1000 fitness scores, and the patients were classified by the 3rd quantile value of each counted neoantigen load. Comparison of differential neoantigen load in all genes (gray line in B), high fitness effect genes (blue bar in B), and low fitness effect genes (red bar in B). Gene Rank indicates the ascending and descending ordered number of genes analyzed for fitness score from bladder cancer cell lines. For this cancer type, we were able to examine fitness effects only because single-cell transcriptomic data were not available.

**Figure S8.** Comparison of differential neoantigen load and patient survival based on fitness scores of genes in the ICI-treated renal cell carcinoma (RCC) cohort.

The KM plot for progression-free survival (PFS) of RCC ICI patients [10] according to high fitness (A, middle), low fitness (A, right), and all genes (A, left). The fitness genes were obtained based on the top 500 or bottom 500 fitness scores, and the patients were classified by the median value of each counted neoantigen load. Comparison of differential neoantigen load in all genes (gray line in B), high fitness effect genes (blue bar in B), and low fitness effect genes (red bar in B). Gene rank indicates the ascending and descending ordered number of genes analyzed for fitness score from RCC cell lines. Statistical significance was not achieved due to the small sample size. For this cancer type, we were able to examine fitness effects only because single-cell transcriptomic data were not available. Also, the differential neoantigen load showed marginal significance because the majority of the RCC group has been known as immune-cold tumor. It was reported that immune surveillance may not occur adequately despite of presentation of immunogenic neoantigens when tumor microenvironment is under immune suppressive conditions as likely in cold tumors [46].

**Figure S9.** Predictions of ICI responses based on neoantigens using **l**ogistic regression classifier.

The ICI responses of individual ICI cohorts of lung cancer (A) and melanoma (B) were predicted with constitutive (blue bars), facultative (red bars), and original neoantigens (gray bars) using a logistic regression classifier. The odds ratio (OR) was obtained corresponding to the 500 genes with the highest and lowest fitness effect scores in individual ICI cohorts of lung cancer and melanoma. OR indicates the likelihood that a clinical response will occur. We used the function of generalized linear model (GLM) implemented in R (glm(family=‘binomial’)).

**Figure S10.** Simulation tests for the normalized neoantigen difference.

All patients in multiple lung cancer (A) and melanoma (B) ICI cohorts were tested for their statistical significances of normalized neoantigen differences by permuting the genes used for the analyses in Figure 3. Same numbers of genes were permuted for each gene rank test and p value was calculated from the distribution of 1,000 normalized neoantigen differences. Grey bar indicates the statistical significance of the normalized neoantigen difference in the case using all genes.

**Figure S11**. Survival analysis of ICI cohorts according to RNA expression.

The KM plot for overall survival (OS) of all patients from the MSK pancancer ICI cohort based on RNA expression is shown. The groups with high or low neoantigen loads were determined by the top (A, left) and bottom (A, right) 500 genes from the sorted RNA expression level – average RNA-seq FPKM value of all tumor tissues in TCGA pancancer cohort. The KM plot for progression-free survival (PFS) of combined lung cancer ICI cohorts based on high (B, left) or low (B, right) RNA expression from TCGA lung cancer patients is shown. The KM plot for the OS of melanoma ICI patients according to high (C, left) or low (C, right) RNA expression from TCGA melanoma patients is shown.

**Figure S12.** The test performance of the DNN model trained on the patient-specific fitness effect in lung cancer.

**Figure S13.** Survival analysis of TCGA patients based on DNN-estimated patient-specific fitness effects between immune clusters

Bar chart of differential neoantigen load between long-term and short-term survivors of TCGA lung cancer and breast cancer patients with hot tumor-enriched immune cluster (A and B, left) and cold tumor-enriched immune cluster (A and B, right). The fitness effect of neoantigens was defined based on the score predicted by the DNN model. The 200 ~ 2000 genes with the highest and lowest DNN-estimated vulnerability of each patient were used to calculate the differential neoantigen load. Forest plot demonstrating hazard ratios for overall survival according to the 500 genes with the highest and lowest fitness scores from DNN model prediction, cell line-based screening, and patient-specific basal expression in TCGA lung cancer (C) and breast cancer patients (D).

**Figure S14.** Simulation tests for the normalized neoantigen difference of TCGA patients.

All patients in multiple lung cancer (A, C) and breast cancer (B, D) cohorts were tested for their statistical significances of normalized neoantigen differences by permuting the genes used for the analyses in Figure 4 and Figure S13. Same numbers of genes were permuted for each gene rank test and p value was calculated from the distribution of 1,000 normalized neoantigen differences. Grey bar indicates the statistical significance of the normalized neoantigen difference in the case using all genes.

**References**

1. Samstein RM, Lee C-H, Shoushtari AN, et al (2019) Tumor mutational load predicts survival after immunotherapy across multiple cancer types. Nat Genet. https://doi.org/10.1038/s41588-018-0312-8

2. Shim JH, Kim HS, Cha H, et al (2020) HLA-corrected tumor mutation burden and homologous recombination deficiency for the prediction of response to PD-(L)1 blockade in advanced non-small-cell lung cancer patients. Ann Oncol 31:902–911. https://doi.org/10.1016/j.annonc.2020.04.004

3. Hellmann MD, Nathanson T, Rizvi H, et al (2018) Genomic Features of Response to Combination Immunotherapy in Patients with Advanced Non-Small-Cell Lung Cancer. Cancer Cell 33:843–852. https://doi.org/10.1016/j.ccell.2018.03.018

4. Rizvi NA, Hellmann MD, Snyder A, et al (2015) Mutational landscape determines sensitivity to PD-1 blockade in non-small cell lung cancer. Science 348:124–128. https://doi.org/10.1126/science.aaa1348

5. Van Allen EM, Miao D, Schilling B, et al (2015) Genomic correlates of response to CTLA-4 blockade in metastatic melanoma. Science 350:207–211. https://doi.org/10.1126/science.aad0095

6. Riaz N, Havel JJ, Makarov V, et al (2017) Tumor and Microenvironment Evolution during Immunotherapy with Nivolumab. Cell 171:934–949. https://doi.org/10.1016/j.cell.2017.09.028

7. Snyder A, Makarov V, Merghoub T, et al (2014) Genetic Basis for Clinical Response to CTLA-4 Blockade in Melanoma. N Engl J Med 371:2189–2199. https://doi.org/10.1056/NEJMoa1406498

8. Hugo W, Zaretsky JM, Sun L, et al (2016) Genomic and Transcriptomic Features of Response to Anti-PD-1 Therapy in Metastatic Melanoma. Cell 165:35–44. https://doi.org/10.1016/j.cell.2016.02.065

9. Mariathasan S, Turley SJ, Nickles D, et al (2018) TGFβ attenuates tumour response to PD-L1 blockade by contributing to exclusion of T cells. Nature 554:544–548. https://doi.org/10.1038/nature25501

10. McDermott DF, Huseni MA, Atkins MB, et al (2018) Clinical activity and molecular correlates of response to atezolizumab alone or in combination with bevacizumab versus sunitinib in renal cell carcinoma. Nat Med 24:749–757. https://doi.org/10.1038/s41591-018-0053-3

11. Li H, Durbin R (2009) Fast and accurate short read alignment with Burrows-Wheeler transform. Bioinformatics 25:1754–60. https://doi.org/10.1093/bioinformatics/btp324

12. McKenna A, Hanna M, Banks E, et al (2010) The Genome Analysis Toolkit: a MapReduce framework for analyzing next-generation DNA sequencing data. Genome Res 20:1297–303. https://doi.org/10.1101/gr.107524.110

13. Benjamin D, Sato T, Cibulskis K, et al (2019) Calling Somatic SNVs and Indels with Mutect2. bioRxiv. https://doi.org/10.1101/861054

14. Shukla SA, Rooney MS, Rajasagi M, et al (2015) Comprehensive analysis of cancer-associated somatic mutations in class i HLA genes. Nat Biotechnol 33:1152–1158. https://doi.org/10.1038/nbt.3344

15. Jurtz V, Paul S, Andreatta M, et al (2017) NetMHCpan-4.0: Improved Peptide–MHC Class I Interaction Predictions Integrating Eluted Ligand and Peptide Binding Affinity Data. J Immunol 199:3360–3368. https://doi.org/10.4049/jimmunol.1700893

16. Vaser R, Adusumalli S, Leng SN, et al (2016) SIFT missense predictions for genomes. Nat Protoc 11:1–9. https://doi.org/10.1038/nprot.2015.123

17. Pacini C, Dempster JM, Goncalves E, et al (2020) Integrated cross-study datasets of genetic dependencies in cancer. bioRxiv 2020.05.22.110247. https://doi.org/10.1101/2020.05.22.110247

18. Meyers RM, Bryan JG, McFarland JM, et al (2017) Computational correction of copy number effect improves specificity of CRISPR–Cas9 essentiality screens in cancer cells. Nat Genet 49:1779–1784. https://doi.org/10.1038/ng.3984

19. Dempster J, Rossen J, Kazachkova M, et al (2019) Extracting Biological Insights from the Project Achilles Genome-Scale CRISPR Screens in Cancer Cell Lines. https://doi.org/10.1101/720243

20. Behan FM, Iorio F, Picco G, et al (2019) Prioritization of cancer therapeutic targets using CRISPR–Cas9 screens. Nature. https://doi.org/10.1038/s41586-019-1103-9

21. Tsherniak A, Vazquez F, Montgomery PG, et al (2017) Defining a Cancer Dependency Map. Cell 170:564–576. https://doi.org/10.1016/j.cell.2017.06.010

22. McDonald ER, de Weck A, Schlabach MR, et al (2017) Project DRIVE: A Compendium of Cancer Dependencies and Synthetic Lethal Relationships Uncovered by Large-Scale, Deep RNAi Screening. Cell 170:577-592.e10. https://doi.org/10.1016/j.cell.2017.07.005

23. Marcotte R, Sayad A, Brown KR, et al (2016) Functional Genomic Landscape of Human Breast Cancer Drivers, Vulnerabilities, and Resistance. Cell 164:293–309. https://doi.org/10.1016/j.cell.2015.11.062

24. McFarland JM, Ho Z V., Kugener G, et al (2018) Improved estimation of cancer dependencies from large-scale RNAi screens using model-based normalization and data integration. Nat Commun 9:4610. https://doi.org/10.1038/s41467-018-06916-5

25. Hart T, Moffat J (2016) BAGEL: A computational framework for identifying essential genes from pooled library screens. BMC Bioinformatics. https://doi.org/10.1186/s12859-016-1015-8

26. Hart T, Chandrashekhar M, Aregger M, et al (2015) High-Resolution CRISPR Screens Reveal Fitness Genes and Genotype-Specific Cancer Liabilities. Cell 163:1515–1526. https://doi.org/10.1016/j.cell.2015.11.015

27. Hart T, Brown KR, Sircoulomb F, et al (2014) Measuring error rates in genomic perturbation screens: gold standards for human functional genomics. Mol Syst Biol 10:733. https://doi.org/10.15252/msb.20145216

28. Jung KC, Sang CK, Seo J, et al (2007) Impact of transcriptional properties on essentiality and evolutionary rate. Genetics 175:199–206. https://doi.org/10.1534/genetics.106.066027

29. Fraser HB, Hirsh AE, Giaever G, et al (2004) Noise Minimization in Eukaryotic Gene Expression. PLoS Biol 2:e137. https://doi.org/10.1371/journal.pbio.0020137

30. Wang Z, Zhang J (2011) Impact of gene expression noise on organismal fitness and the efficacy of natural selection. Proc Natl Acad Sci U S A 108:. https://doi.org/10.1073/pnas.1100059108

31. Patel AP, Tirosh I, Trombetta JJ, et al (2014) Single-cell RNA-seq highlights intratumoral heterogeneity in primary glioblastoma. Science 344:1396–1401. https://doi.org/10.1126/science.1254257

32. Karaayvaz M, Cristea S, Gillespie SM, et al (2018) Unravelling subclonal heterogeneity and aggressive disease states in TNBC through single-cell RNA-seq. Nat Commun 9:3588. https://doi.org/10.1038/s41467-018-06052-0

33. Fan J, Lee H-O, Lee S, et al (2018) Linking transcriptional and genetic tumor heterogeneity through allele analysis of single-cell RNA-seq data. Genome Res 28:1217–1227. https://doi.org/10.1101/gr.228080.117

34. Ferrall-Fairbanks MC, Ball M, Padron E, Altrock PM (2019) Leveraging Single-Cell RNA Sequencing Experiments to Model Intratumor Heterogeneity. JCO Clin Cancer Informatics 1–10. https://doi.org/10.1200/cci.18.00074

35. Kim N, Kim HK, Lee K, et al (2020) Single-cell RNA sequencing demonstrates the molecular and cellular reprogramming of metastatic lung adenocarcinoma. Nat Commun 11:. https://doi.org/10.1038/s41467-020-16164-1

36. Jerby-Arnon L, Shah P, Cuoco MS, et al (2018) A Cancer Cell Program Promotes T Cell Exclusion and Resistance to Checkpoint Blockade. Cell 175:984-997.e24. https://doi.org/10.1016/j.cell.2018.09.006

37. O’Leary NA, Wright MW, Brister JR, et al (2016) Reference sequence (RefSeq) database at NCBI: Current status, taxonomic expansion, and functional annotation. Nucleic Acids Res 44:D733–D745. https://doi.org/10.1093/nar/gkv1189

38. Suzuki A, Matsushima K, Makinoshima H, et al (2015) Single-cell analysis of lung adenocarcinoma cell lines reveals diverse expression patterns of individual cells invoked by a molecular target drug treatment. Genome Biol 16:1–17. https://doi.org/10.1186/s13059-015-0636-y

39. Roth A, Khattra J, Yap D, et al (2014) PyClone: statistical inference of clonal population structure in cancer. Nat Methods 11:396–398. https://doi.org/10.1038/nmeth.2883

40. Goldman, M.J., Craft, B., Hastie M et al. (2020) Visualizing and interpreting cancer genomics data via the Xena platform. Nat Biotechnol 38:678–678. https://doi.org/10.1038/s41587-020-0546-8

41. Kim K, Yang W, Lee KS, et al (2015) Global transcription network incorporating distal regulator binding reveals selective cooperation of cancer drivers and risk genes. Nucleic Acids Res 43:5716–5729. https://doi.org/10.1093/nar/gkv532

42. Jang K, Park MJ, Park JS, et al (2020) Computational inference of cancer-specific vulnerabilities in clinical samples. Genome Biol 21:155. https://doi.org/10.1186/s13059-020-02077-1

43. Lachmann A, Giorgi FM, Lopez G, Califano A (2016) ARACNe-AP: Gene network reverse engineering through adaptive partitioning inference of mutual information. Bioinformatics 32:2233–2235. https://doi.org/10.1093/bioinformatics/btw216

44. Aran D, Hu Z, Butte AJ (2017) xCell: digitally portraying the tissue cellular heterogeneity landscape. Genome Biol 18:220. https://doi.org/10.1186/s13059-017-1349-1

45. Monti S, Tamayo P, Mesirov J, Golub T (2003) Consensus Clustering: A Resampling-Based Method for Class Discovery and Visualization of Gene Expression Microarray Data. Mach Learn 52:91–118. https://doi.org/10.1023/A:1023949509487

46. Nicholas M, Charles S (2019) Neoantigen quality, not quantity. Sci Transl Med 11:eaax7918. https://doi.org/10.1126/scitranslmed.aax7918
