# Supplementary material for: Gene essentiality for tumour growth influences neoantigen‐directed immunoediting
Source: Clin Transl Med. 2022 Jan 21;12(1):e714. doi: 10.1002/ctm2.714 (PMC8778643; doi:10.1002/ctm2.714)
Supplement: Supplementary file 3 — Supporting Information [file CTM2-12-e714-s003.pdf]

Figure S1

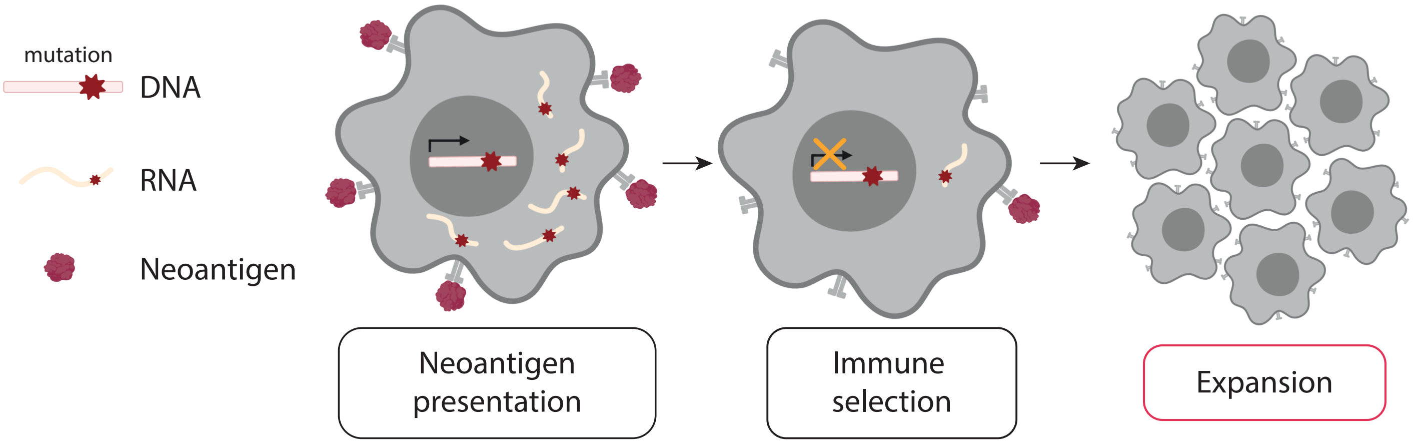

mutation
DNA
RNA
Neoantigen
Neoantigen presentation
Immune selection
Expansion

Figure S2

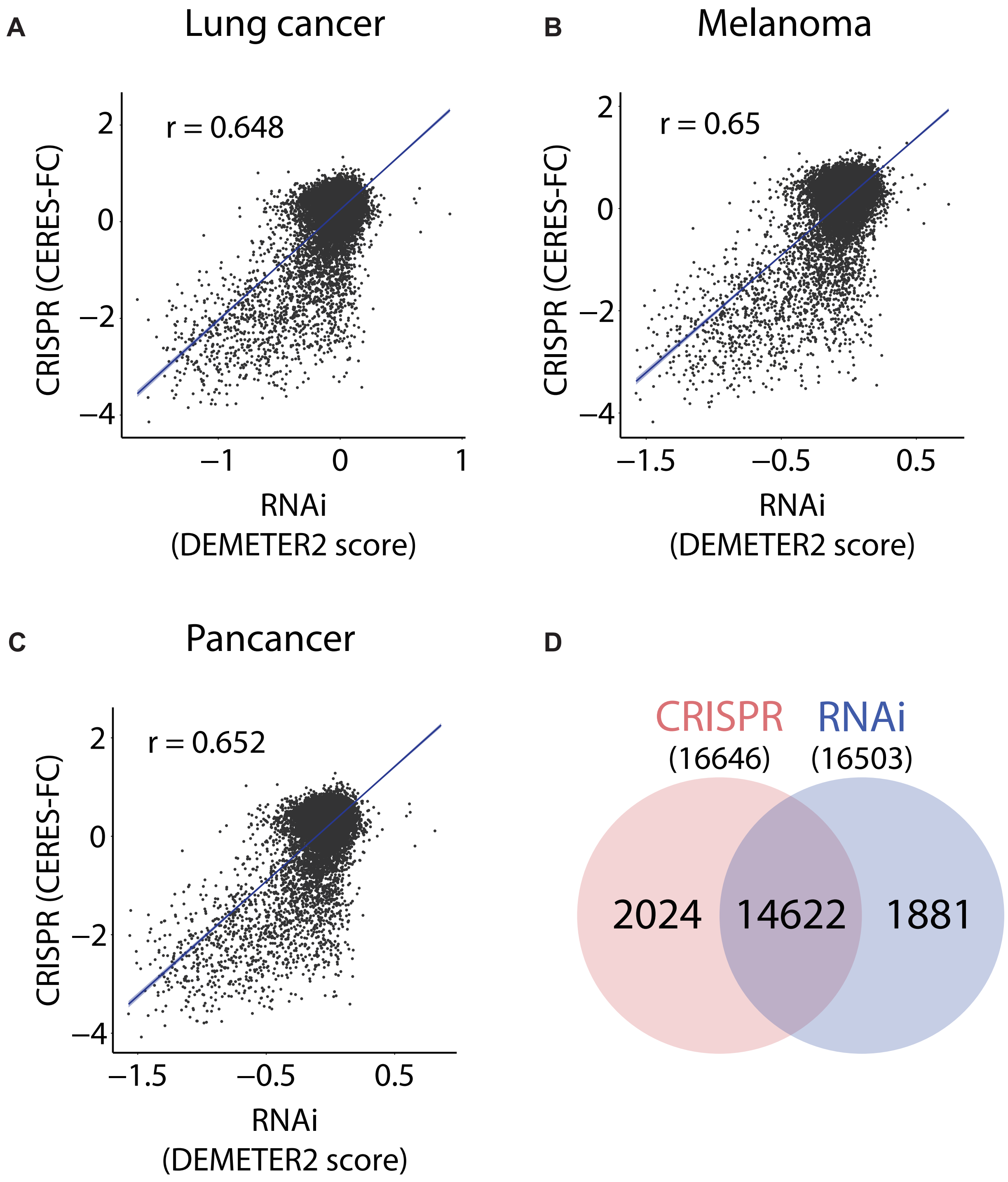
A
Lung cancer
r = 0.648
CRISPR (CERES-FC)
2
0
−2
−4
−1
0
1
RNAi
(DEMETER2 score)
B
Melanoma
r = 0.65
CRISPR (CERES-FC)
2
0
−2
−4
−1.5
−0.5
0.5
RNAi
(DEMETER2 score)
C
Pancancer
r = 0.652
CRISPR (CERES-FC)
2
0
−2
−4
−1.5
−0.5
0.5
RNAi
(DEMETER2 score)
D
CRISPR
(16646)
RNAi
(16503)
2024
14622
1881

Figure S3

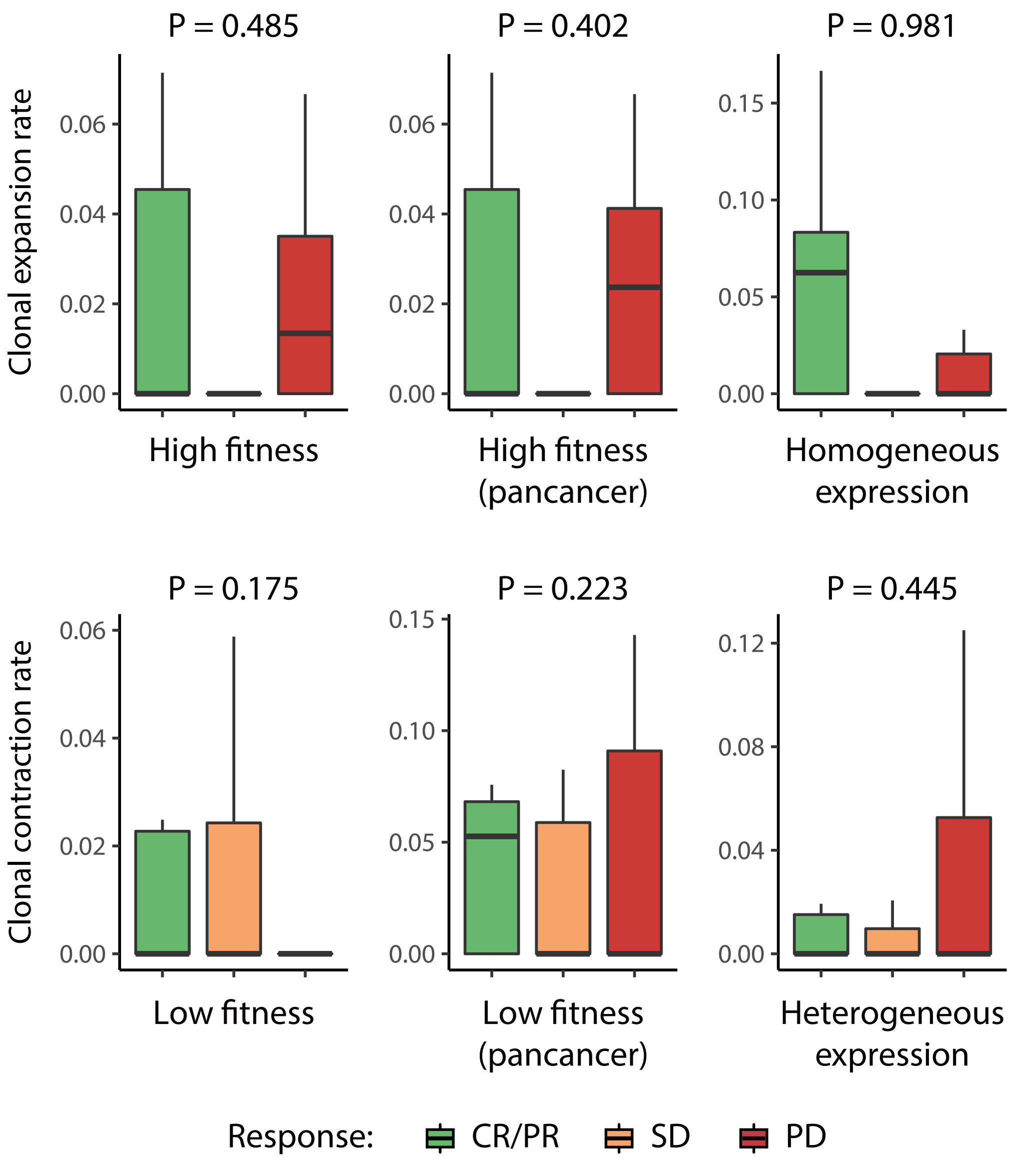
P = 0.485
P = 0.402
P = 0.981
Clonal expansion rate
0.06
0.04
0.02
0.00
0.15
0.10
0.05
High fitness
High fitness (pancancer)
Homogeneous expression
P = 0.175
P = 0.223
P = 0.445
Clonal contraction rate
0.12
0.08
Low fitness
Low fitness (pancancer)
Heterogeneous expression
Response:
CR/PR
SD
PD

Figure S4

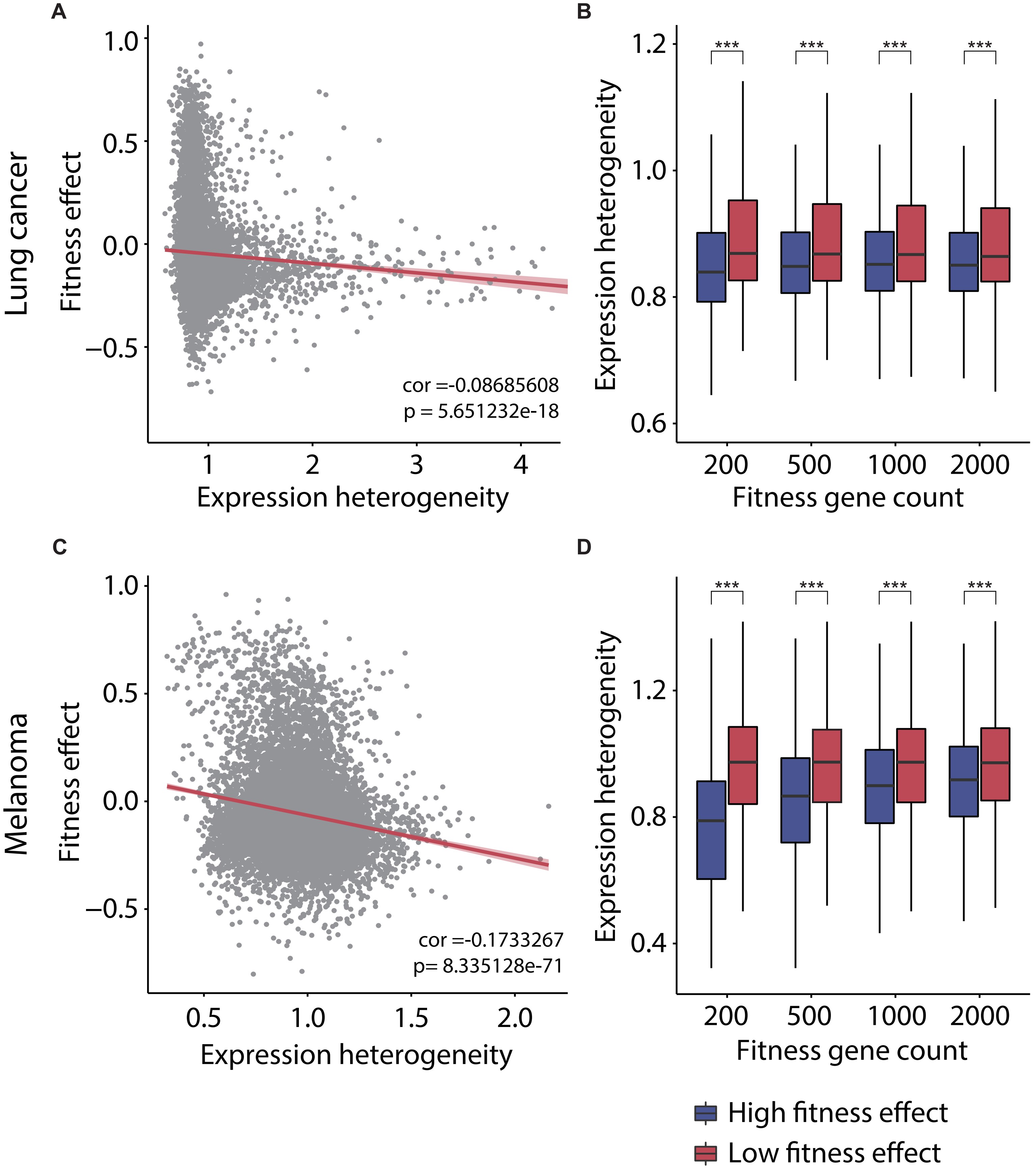
A
B
C
D
Lung cancer
Melanoma
Fitness effect
1.0
0.5
0.0
−0.5
1
2
3
4
Expression heterogeneity
cor =-0.08685608
p = 5.651232e-18
Expression heterogeneity
1.2
1.0
0.8
0.6
***
200
500
1000
2000
Fitness gene count
0.5
1.0
1.5
2.0
cor =-0.1733267
p= 8.335128e-71
0.4
High fitness effect
Low fitness effect

Figure S5

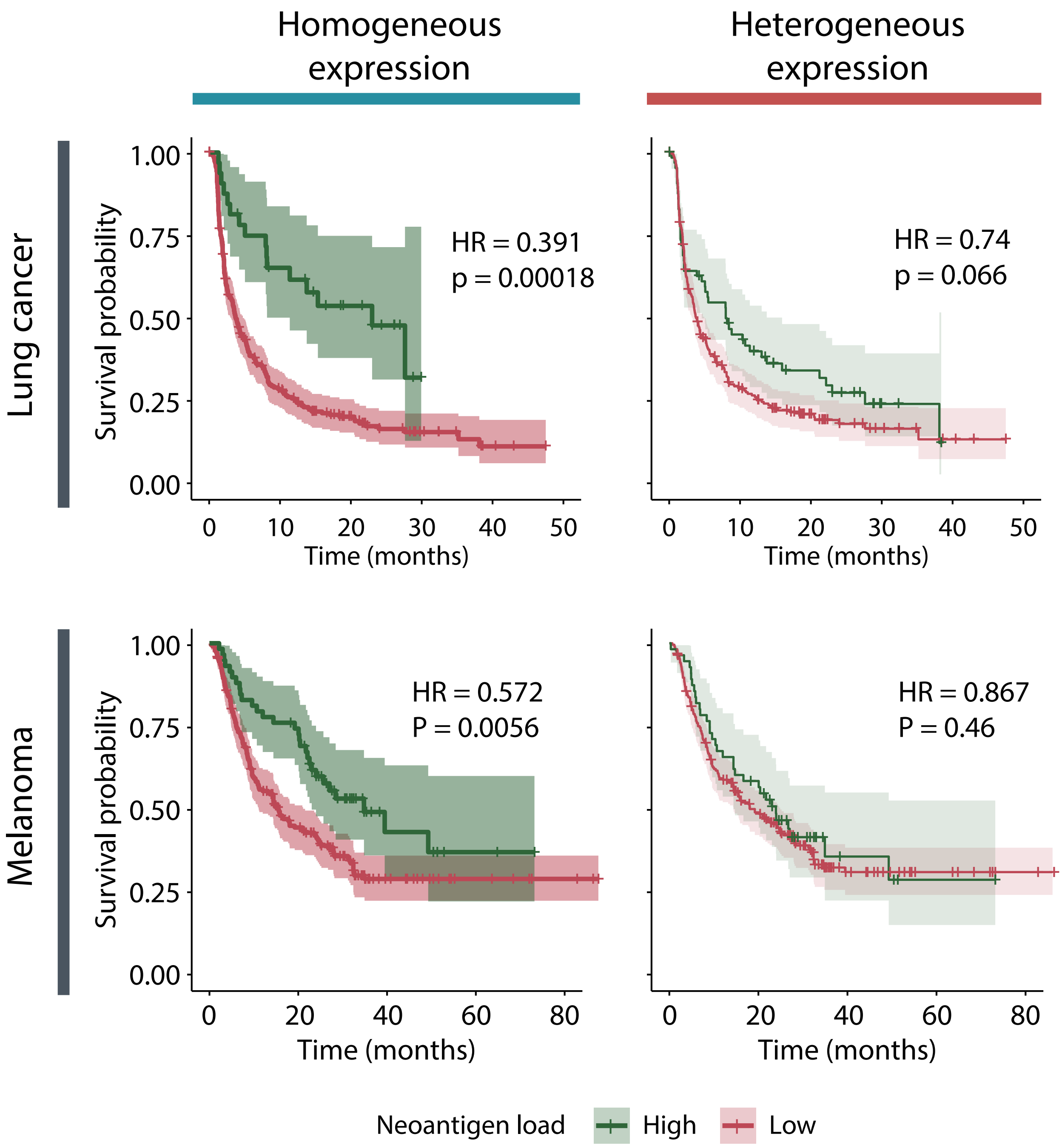

Homogeneous expression
Heterogeneous expression
Lung cancer
Melanoma
Survival probability
1.00
0.75
0.50
0.25
0.00
HR = 0.391
p = 0.00018
HR = 0.74
p = 0.066
HR = 0.572
P = 0.0056
HR = 0.867
P = 0.46
0
10
20
30
40
50
40
60
80
Time (months)
Neoantigen load
High
Low

Figure S6

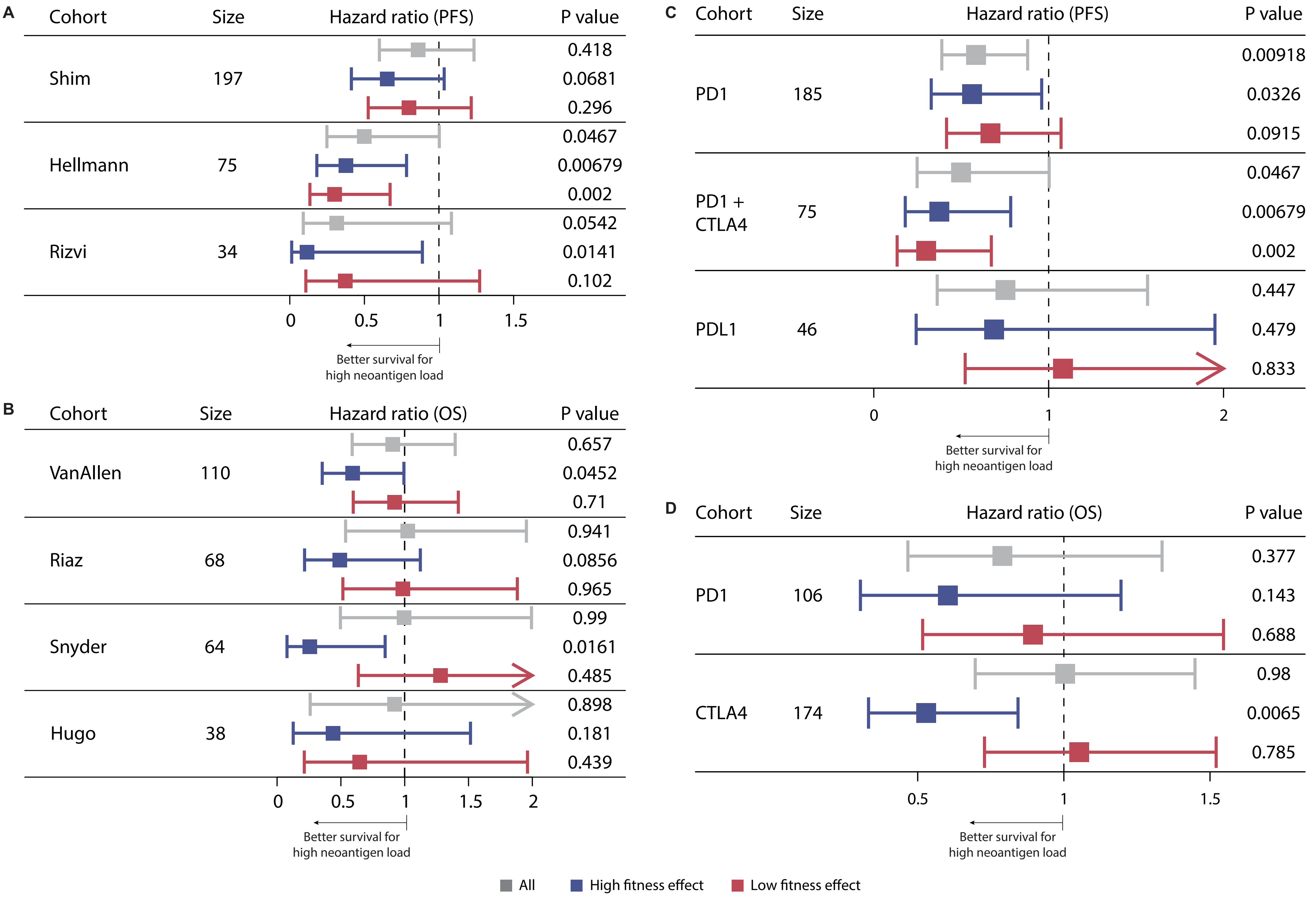

A
Cohort
Size
Hazard ratio (PFS)
P value
Shim
197
0.418
0.0681
0.296
Hellmann
75
0.0467
0.00679
0.002
Rizvi
34
0.0542
0.0141
0.102
0
0.5
1
1.5
Better survival for
high neoantigen load
B
Cohort
Size
Hazard ratio (OS)
P value
VanAllen
110
0.657
0.0452
0.71
Riaz
68
0.941
0.0856
0.965
Snyder
64
0.99
0.0161
0.485
Hugo
38
0.898
0.181
0.439
0
0.5
1
1.5
2
Better survival for
high neoantigen load
C
Cohort
Size
Hazard ratio (PFS)
P value
PD1
185
0.00918
0.0326
0.0915
PD1 +
CTLA4
75
0.0467
0.00679
0.002
PDL1
46
0.447
0.479
0.833
0
1
2
Better survival for
high neoantigen load
D
Cohort
Size
Hazard ratio (OS)
P value
PD1
106
0.377
0.143
0.688
CTLA4
174
0.98
0.0065
0.785
0.5
1
1.5
Better survival for
high neoantigen load
All
High fitness effect
Low fitness effect

Figure S7

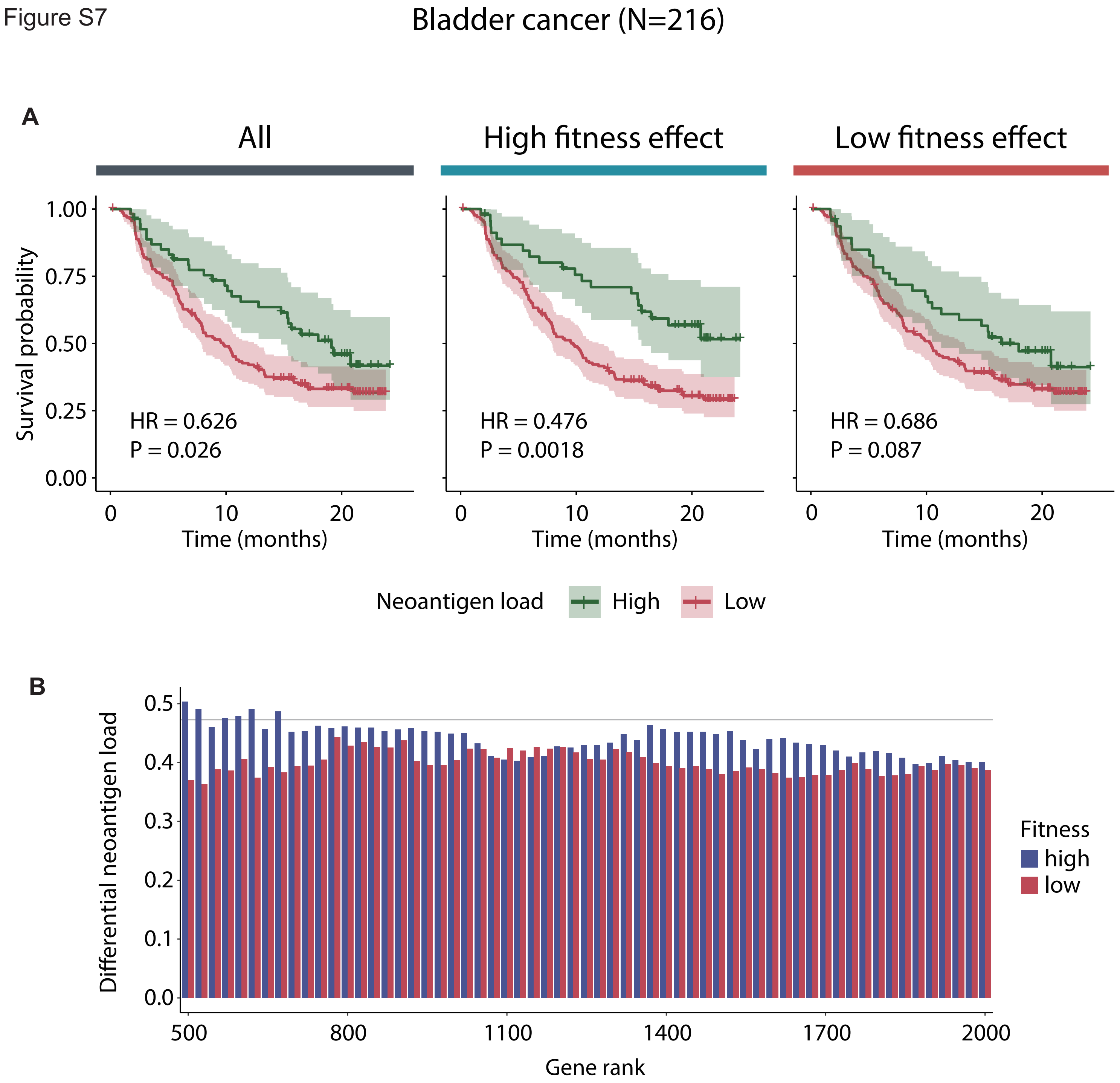

Bladder cancer (N=216)
A
All
High fitness effect
Low fitness effect
Survival probability
1.00
0.75
0.50
0.25
0.00
HR = 0.626
P = 0.026
HR = 0.476
P = 0.0018
HR = 0.686
P = 0.087
0
10
20
Time (months)
Neoantigen load
High
Low
B
Differential neoantigen load
0.5
0.4
0.3
0.2
0.1
0.0
500
800
1100
1400
1700
2000
Gene rank
Fitness
high
low

Figure S8

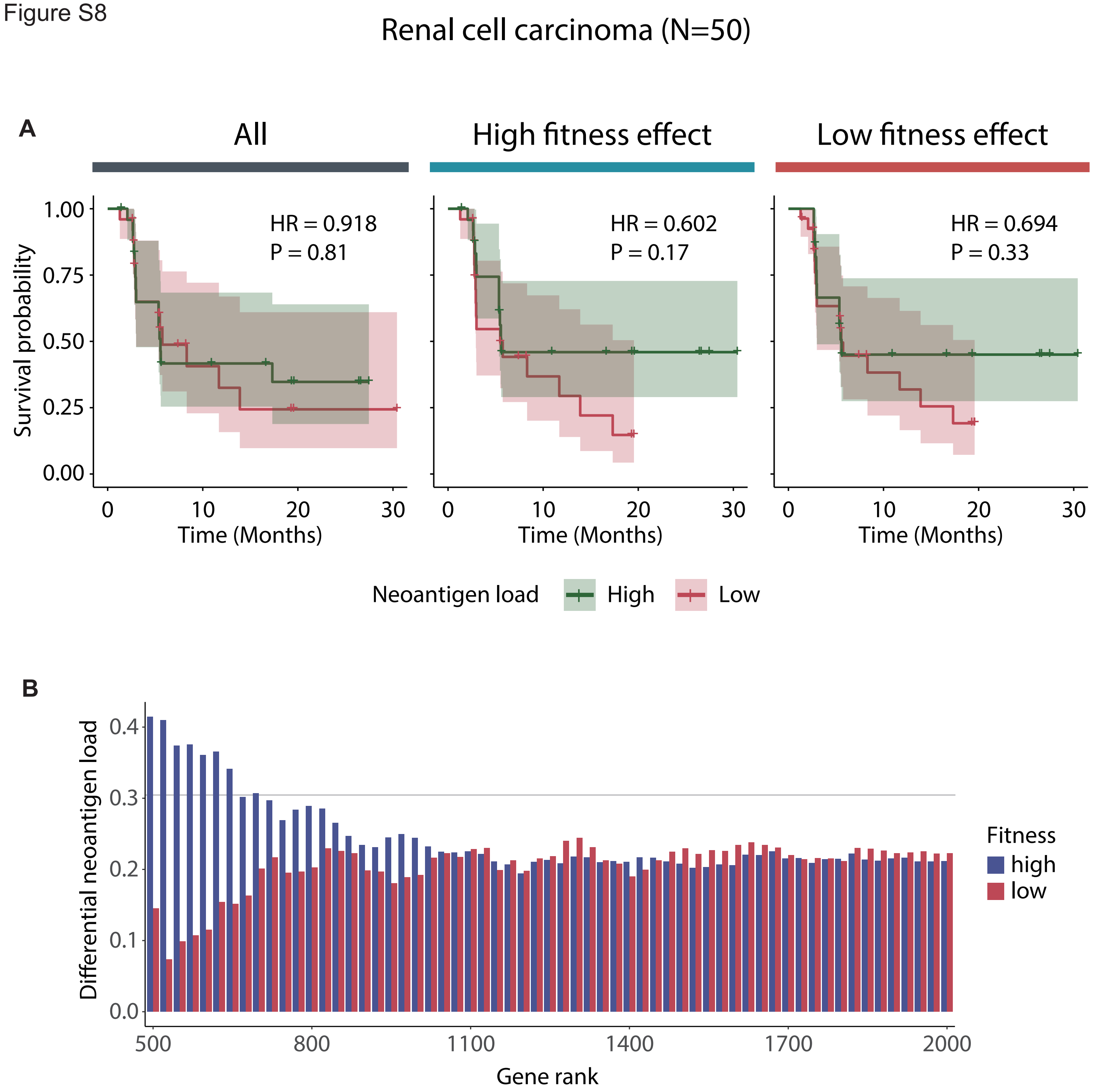
Renal cell carcinoma (N=50)
A
All
High fitness effect
Low fitness effect
HR = 0.918
P = 0.81
HR = 0.602
P = 0.17
HR = 0.694
P = 0.33
Survival probability
1.00
0.75
0.50
0.25
0.00
0
10
20
30
Time (Months)
Neoantigen load
High
Low
B
Differential neoantigen load
0.4
0.3
0.2
0.1
0.0
500
800
1100
1400
1700
2000
Gene rank
Fitness
high
low

Figure S9

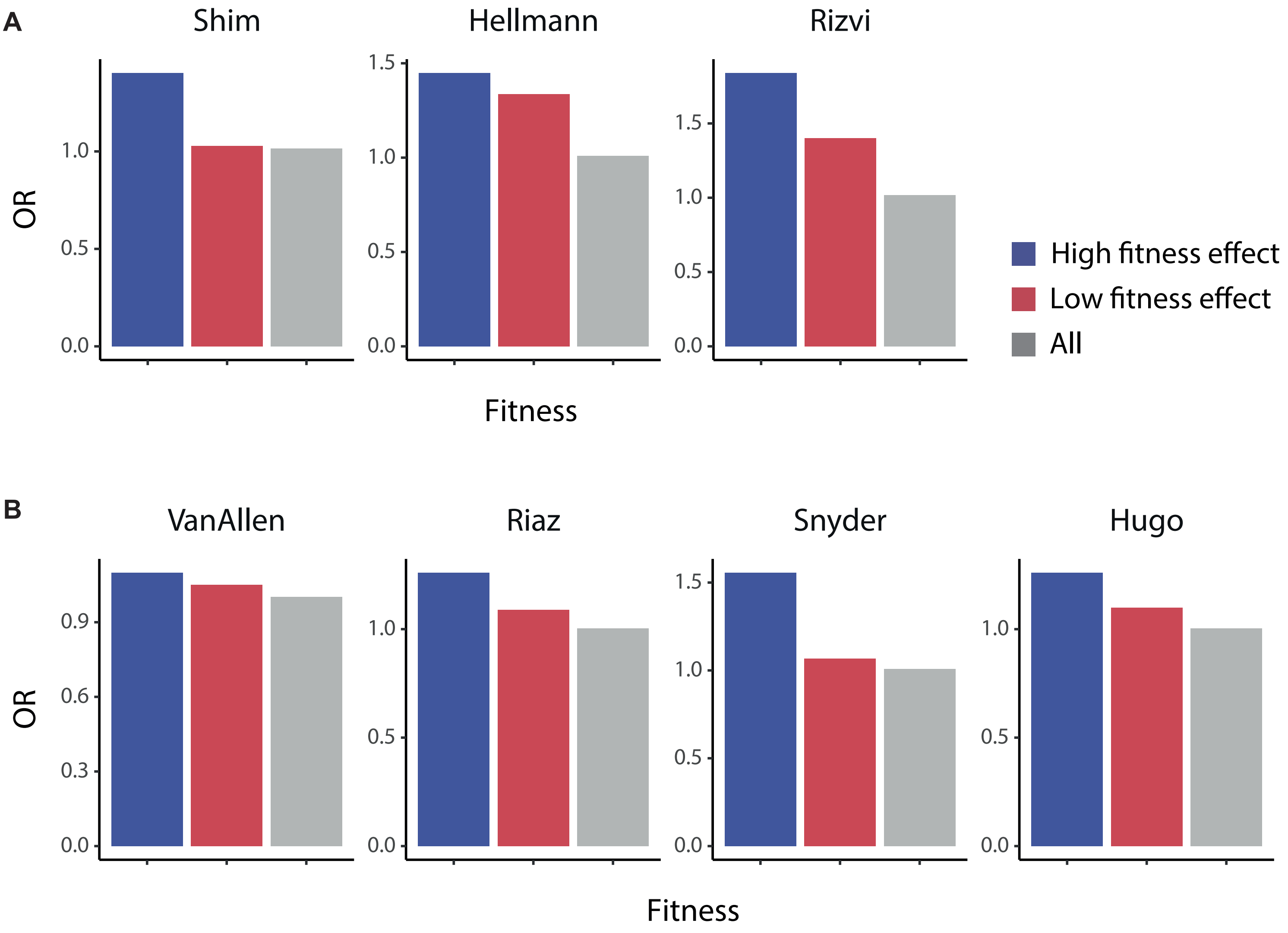

A
Shim
Hellmann
Rizvi
OR
1.5
1.0
0.5
0.0
Fitness
High fitness effect
Low fitness effect
All
B
VanAllen
Riaz
Snyder
Hugo
OR
0.9
0.6
0.3
0.0
Fitness

Figure S10

A

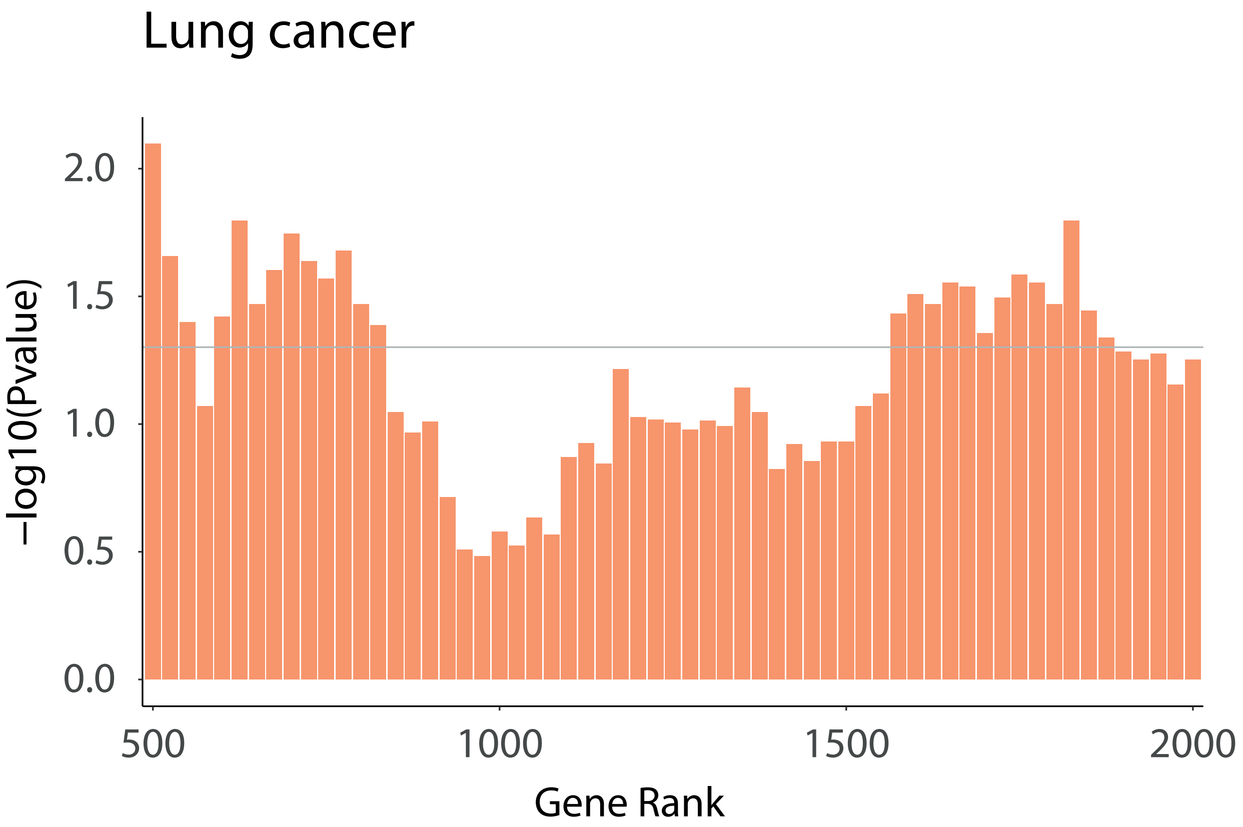

B

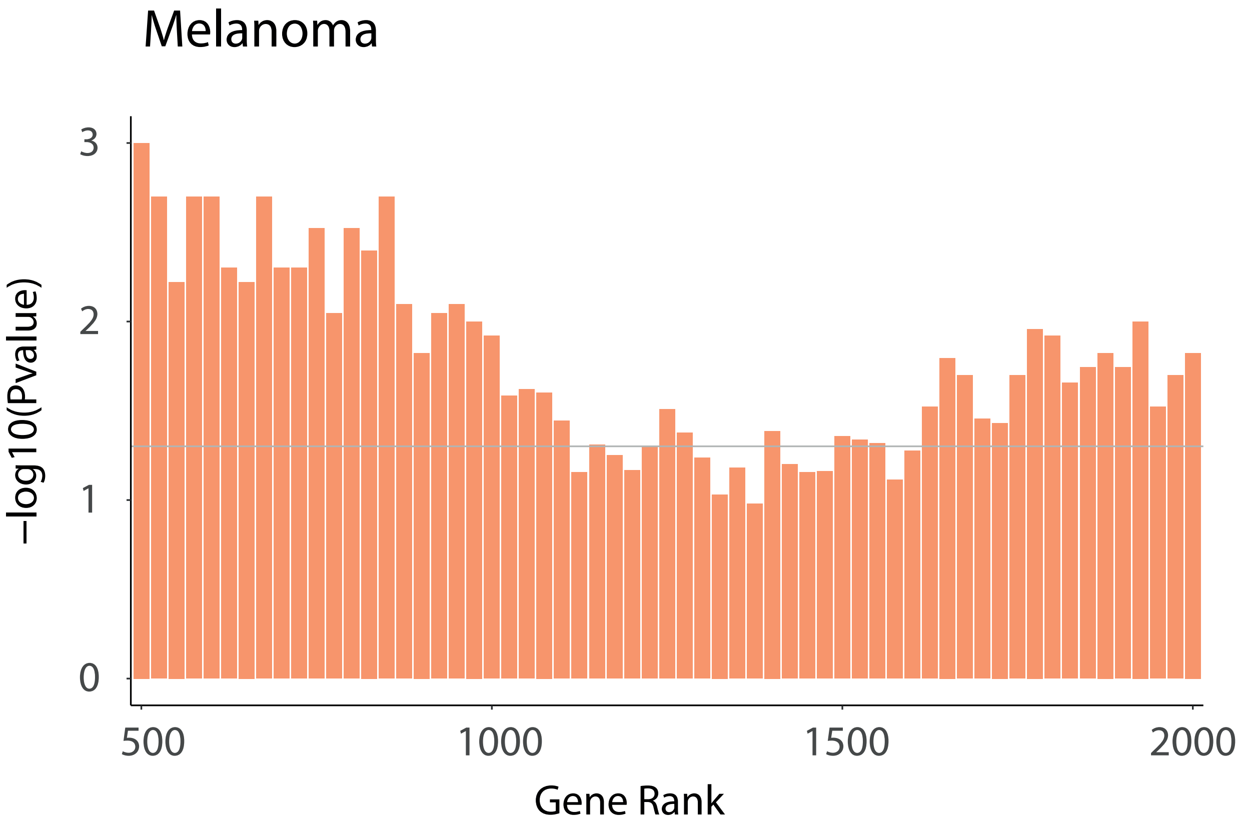

Figure S11

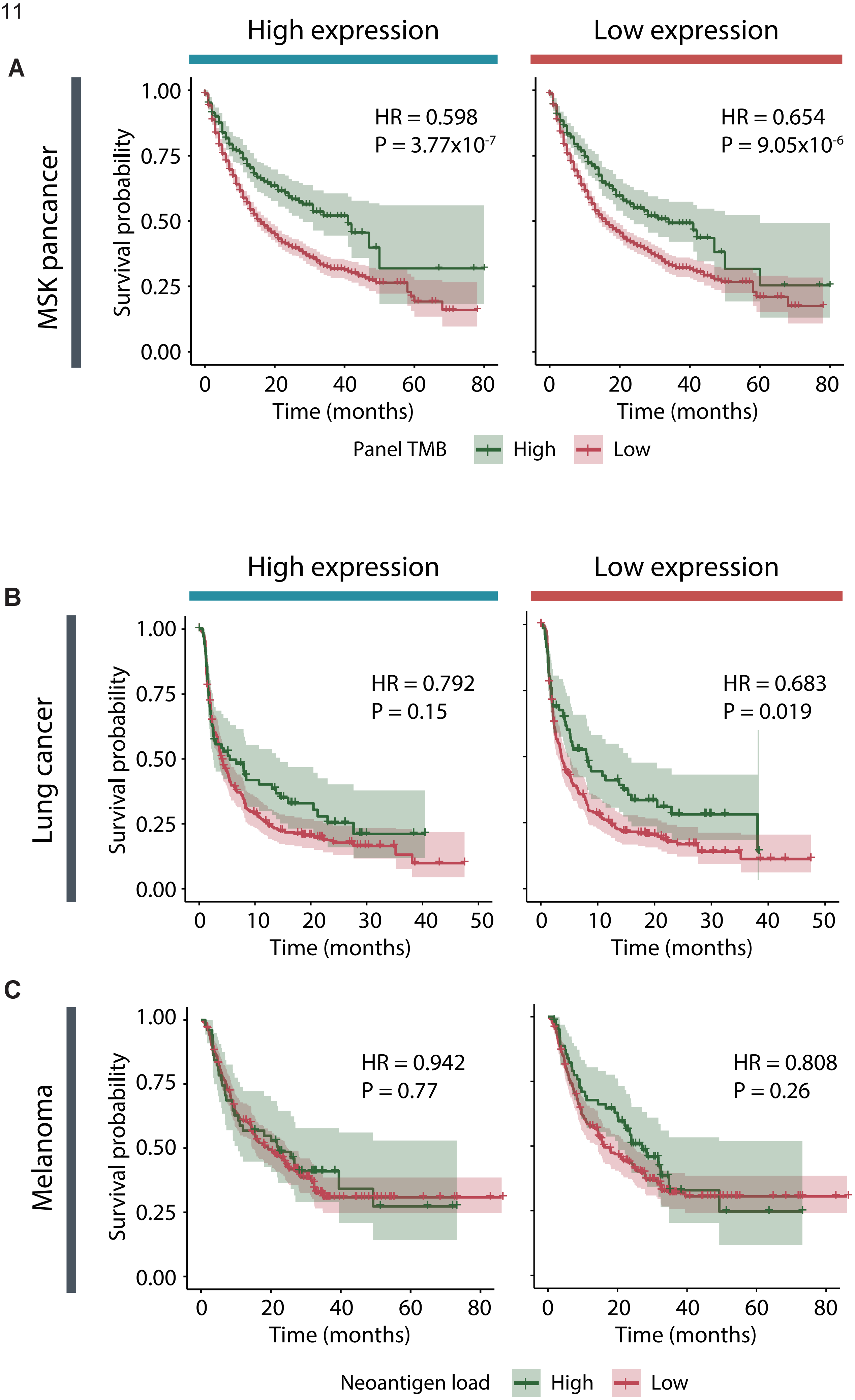

A
B
C
High expression
Low expression
MSK pancancer
Lung cancer
Melanoma
Survival probability
1.00
0.75
0.50
0.25
0.00
Time (months)
0
10
20
30
40
50
60
80
HR = 0.598
P = 3.77x10-7
HR = 0.654
P = 9.05x10-6
Panel TMB
High
Low
HR = 0.792
P = 0.15
HR = 0.683
P = 0.019
HR = 0.942
P = 0.77
HR = 0.808
P = 0.26
Neoantigen load

Figure S12

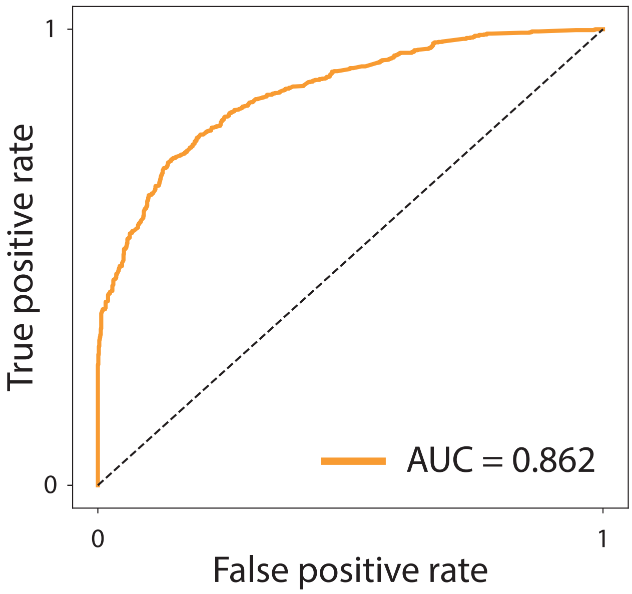

Figure S13

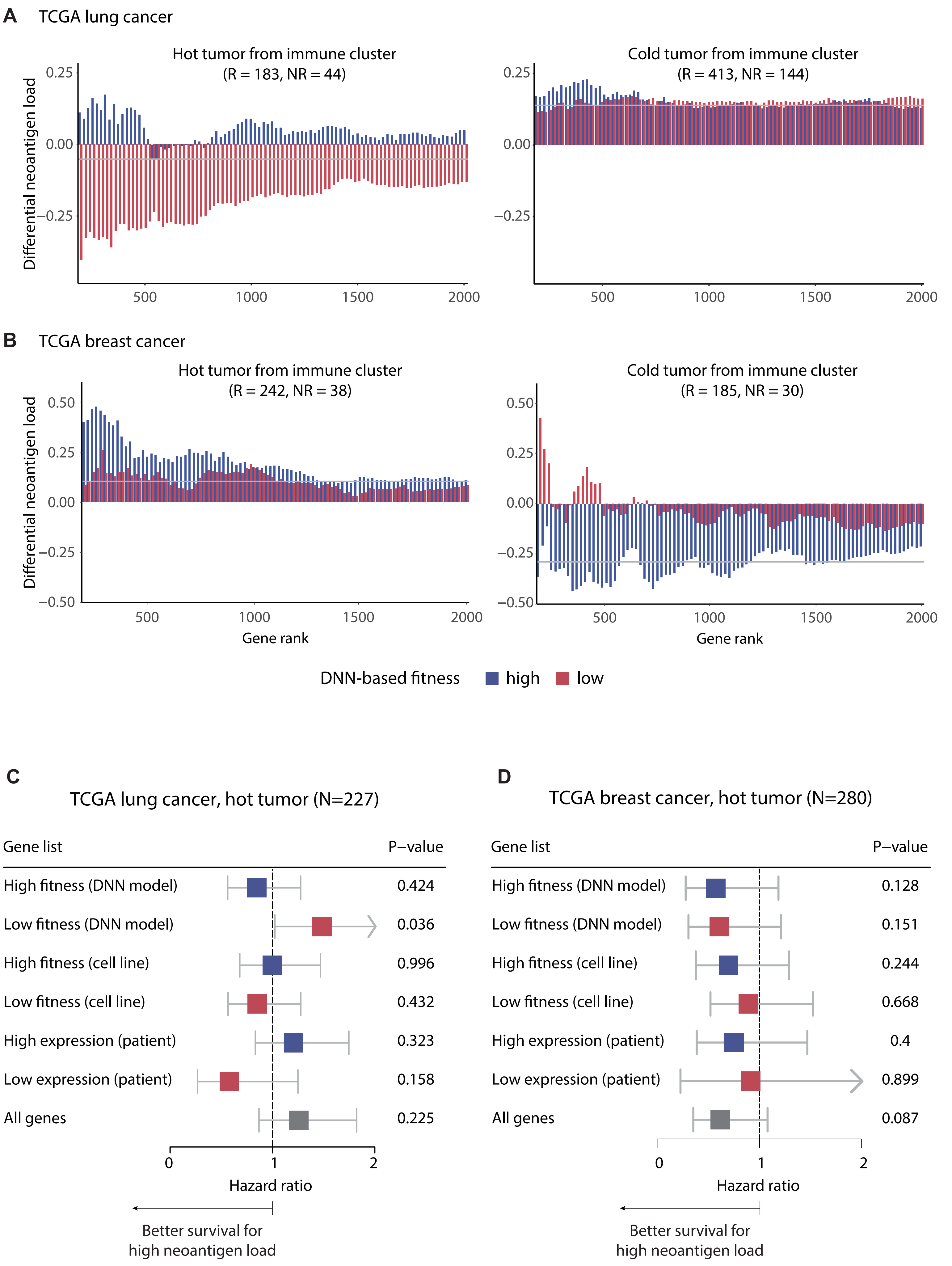
A
TCGA lung cancer
Hot tumor from immune cluster
(R = 183, NR = 44)
Cold tumor from immune cluster
(R = 413, NR = 144)
Differential neoantigen load
0.25
0.00
−0.25
500
1000
1500
2000
B
TCGA breast cancer
Hot tumor from immune cluster
(R = 242, NR = 38)
Cold tumor from immune cluster
(R = 185, NR = 30)
Differential neoantigen load
0.50
0.25
0.00
−0.25
−0.50
Gene rank
DNN-based fitness
high
low
C
TCGA lung cancer, hot tumor (N=227)
Gene list
P−value
High fitness (DNN model) 0.424
Low fitness (DNN model) 0.036
High fitness (cell line) 0.996
Low fitness (cell line) 0.432
High expression (patient) 0.323
Low expression (patient) 0.158
All genes 0.225
0
1
2
Hazard ratio
Better survival for
high neoantigen load
D
TCGA breast cancer, hot tumor (N=280)
Gene list
P−value
High fitness (DNN model) 0.128
Low fitness (DNN model) 0.151
High fitness (cell line) 0.244
Low fitness (cell line) 0.668
High expression (patient) 0.4
Low expression (patient) 0.899
All genes 0.087
0
1
2
Hazard ratio
Better survival for
high neoantigen load

Figure S14

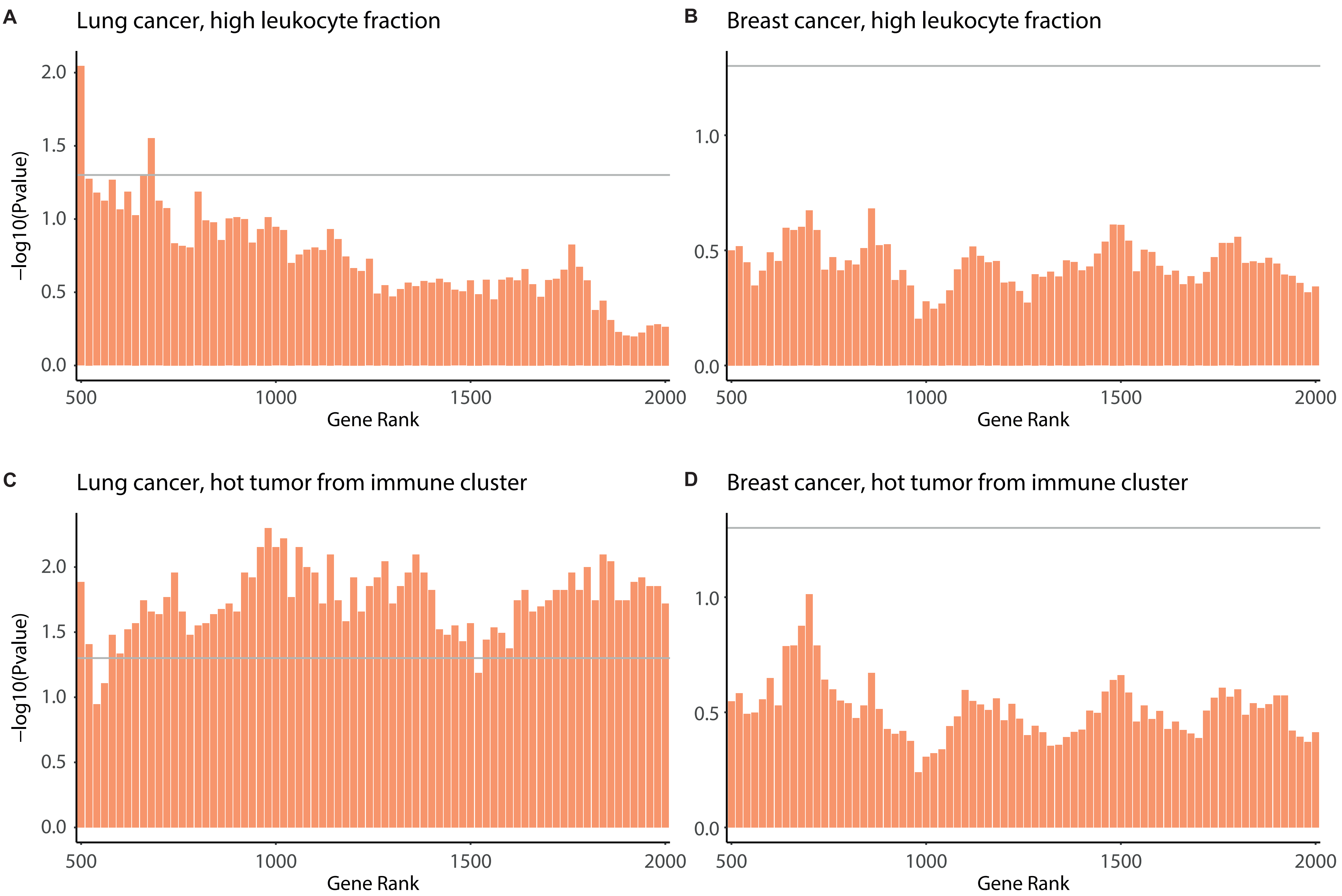
A
Lung cancer, high leukocyte fraction
−log10(Pvalue)
2.0
1.5
1.0
0.5
0.0
500
1000
1500
2000
Gene Rank
B
Breast cancer, high leukocyte fraction
1.0
0.5
0.0
500
1000
1500
2000
Gene Rank
C
Lung cancer, hot tumor from immune cluster
−log10(Pvalue)
2.0
1.5
1.0
0.5
0.0
500
1000
1500
2000
Gene Rank
D
Breast cancer, hot tumor from immune cluster
1.0
0.5
0.0
500
1000
1500
2000
Gene Rank
